# Disruption of RING and PHD Domains of TRIM28 Evokes Differentiation in Human iPSCs

**DOI:** 10.3390/cells10081933

**Published:** 2021-07-29

**Authors:** Sylwia Mazurek, Urszula Oleksiewicz, Patrycja Czerwińska, Joanna Wróblewska, Marta Klimczak, Maciej Wiznerowicz

**Affiliations:** 1 Department of Cancer Immunology, Chair of Medical Biotechnology, Poznan University of Medical Sciences, 61-701 Poznan, Poland; oleksiewiczu@ump.edu.pl (U.O.); czerwinska.patrycja@ump.edu.pl (P.C.); maciej.wiznerowicz@iimo.pl (M.W.); 2 Department of Cancer Diagnostics and Immunology, Greater Poland Cancer Centre, 61-866 Poznan, Poland; 3 Department of Tumor Pathology and Prophylaxis, Poznan University of Medical Sciences, 61-701 Poznan, Poland; jpwroblewska@ump.edu.pl; 4 Department of Tumor Pathology, Greater Poland Cancer Centre, 61-866 Poznan, Poland; 5 International Institute of Molecular and Cell Biology, 02-109 Warsaw, Poland; m.klimczak01@gmail.com; 6 International Institute for Molecular Oncology, 60-203 Poznan, Poland

**Keywords:** TRIM28, KAP1, iPS, stem cells, stemness, pluripotency, self-renewal, epigenetics, differentiation, cancer

## Abstract

TRIM28, a multi-domain protein, is crucial in the development of mouse embryos and the maintenance of embryonic stem cells’ (ESC) self-renewal potential. As the epigenetic factor modulating chromatin structure, TRIM28 regulates the expression of numerous genes and is associated with progression and poor prognosis in many types of cancer. Because of many similarities between highly dedifferentiated cancer cells and normal pluripotent stem cells, we applied human induced pluripotent stem cells (hiPSC) as a model for stemness studies. For the first time in hiPSC, we analyzed the function of individual TRIM28 domains. Here we demonstrate the essential role of a really interesting new gene (RING) domain and plant homeodomain (PHD) in regulating pluripotency maintenance and self-renewal capacity of hiPSC. Our data indicate that mutation within the RING or PHD domain leads to the loss of stem cell phenotypes and downregulation of the FGF signaling. Moreover, impairment of RING or PHD domain results in decreased proliferation and impedes embryoid body formation. In opposition to previous data indicating the impact of phosphorylation on TRIM28 function, our data suggest that TRIM28 phosphorylation does not significantly affect the pluripotency and self-renewal maintenance of hiPSC. Of note, iPSC with disrupted RING and PHD functions display downregulation of genes associated with tumor metastasis, which are considered important targets in cancer treatment. Our data suggest the potential use of RING and PHD domains of TRIM28 as targets in cancer therapy.

## 1. Introduction

Due to self-renewal and pluripotency maintenance properties, induced pluripotent stem cells (iPSC) exhibit several features that are also characteristic for cancer cells, e.g., the similar expression profile of many genes or activity of signaling pathways regulating self-renewal [1,2,3]. Epigenetic and transcriptional dysregulations in tumor cells disturb many signaling pathways also responsible for maintaining the phenotype of normal stem cells, leading to progressive dedifferentiation and acquisition of stemness features [2,3]. The stemness score is the lowest in somatic cells, increased in primary tumors, and reaches the highest level in tumor metastases. This indicates that tumor progression usually involves the process of oncogenic dedifferentiation [3]. Because of many similarities, iPSC can contribute to developing strategies for inhibiting the oncogenic switch of primary tumor cells to more malignant cells found in metastases.

A number of different mechanisms determine the ability of stem cells to maintain their phenotype, and many of them play important roles in embryonic, somatic, induced, and cancer stem cells [2,3,4,5,6]. These mechanisms include numerous signaling pathways and the core pluripotency network comprised of OCT3/4, SOX2, and NANOG. The conversion between stemness and differentiation also depends on more complex epigenetic and non-epigenetic gene expression regulation.

We and others showed that tripartite motif-containing protein 28 (TRIM28) is implicated in the epigenetic control of genes responsible for maintaining stemness and self-renewal [7,8,9,10]. TRIM28, also known as KAP1 (KRAB (Krüppel associated box)-associated protein 1)), or TIF1β (transcription intermediary factor 1-beta), is a multi-domain protein, modulating the structure of chromatin [11,12]. It is highly expressed in embryonic stem cells (ESC) and is crucial in embryogenesis and gastrulation in mice [13,14]. Trim28 is indispensable for the reprogramming of mouse fibroblasts into iPSC [15] and in stemness maintenance in mouse and human PSC [8,16,17,18], as well as in human cancer cells [9,19]. Several studies indicated a positive correlation between TRIM28 upregulation and poor cancer prognosis in specific tumor types [20,21,22,23,24,25].

Each of the TRIM28 protein domains performs a separate, strictly defined function (Graphical Abstract, Figure S1, Table S1). At the N-terminus, TRIM28 contains the tripartite motif (TRIM) responsible for protein–protein interaction and oligomerization. The TRIM motif consists of three sub-units: the really interesting new gene (RING) finger, two B-box zinc fingers, and the coiled-coil (CC) motif, altogether described as RING-B-box-Coiled-Coil (RBCC) domain [11]. The RBCC domain interacts with the KRAB domain of epigenetic repressors—KRAB zinc-finger proteins (KRAB-ZFPs) [11]. In the central part, TRIM28 contains the heterochromatin protein 1-binding domain (HP1BD) [26]. HP1BD recruits HP1 protein to activate heterochromatization. The C-terminus of TRIM28 contains the plant homeodomain (PHD) and bromodomain (BROMO) functional unit [27]. PHD mediates the sumoylation of adjacent BROMO [28,29]. This induces local chromatin concentration by recruiting SET domain bifurcated 1 histone lysine methyltransferase (SETDB1) specific to H3K9 and the nucleosome remodeling and deacetylation (NuRD) complex [30]. It is considered that PHD, BROMO, and the PxVxL motif present in the HP1BD domain [31] work in cooperation to initiate heterochromatin formation, which is characterized by low histone acetylation, increased H3K9me3, and binding of HP1 [30].

Due to the numerous common features between highly dedifferentiated cancer cells and normal pluripotent stem cells, and, above all, similarities in stemness and self-renewal maintenance, we applied human iPSC as a model for stemness studies. Here we show that cells lacking TRIM28 lose the expression of pluripotency markers, as well as the ability to self-renew, and they start to differentiate. Moreover, we identified two TRIM28 domains, RING and PHD, responsible for iPSC’s self-renewal capacity and pluripotency maintenance. Mutation of RING or PHD resulted in the downregulation of pluripotency markers and proliferation, self-renewal inhibition, and restriction in embryoid bodies (EBs) formation. Finally, we found that mutation in the RING or PHD domain caused the downregulation of genes frequently associated with metastasis or poor prognosis in cancer patients. Altogether, our observations indicate that TRIM28 maintains the state of pluripotency and self-renewal by its transcriptional co-repressor activity via RING and PHD.

## 2. Materials and Methods

### 2.1. Organisms

Immunodeficient NUDE mice, NOD.CB17-Prkdc^scid^/NCrCrl (RRID:IMSR_CRL:394, Charles River Laboratories, Wilmington, MA, USA) were kept in the animal rooms of the Institute of Human Genetics, Polish Academy of Sciences, Poznan, Poland. Cages were equipped with HEPA filters and internal air circulation. The mice were provided with a 12-h day/night cycle and unlimited access to water and food. The temperature in the breeding rooms was regulated automatically.

### 2.2. Cell Culture

PHDF cells were isolated from the skin fragments obtained during mastectomy surgeries performed at the Greater Poland Cancer Centre, Poznań, Poland. Each time, informed patient consent participation in “The Cancer Genome Atlas (TCGA)” project was obtained to use biological material for scientific research. Patient identities were not known to researchers. The skin fragment was cut into smaller sizes and incubated overnight at 37 °C in a digestion medium consisting of DMEM High Glucose (#L0102, Biowest, Nuaillé, France), 3 mg/mL collagenase IV (#17104019, Gibco, Thermo Fisher Scientific, Waltham, MA, USA), and 1% penicillin-streptomycin (#P4333, Sigma-Aldrich, St. Louis, MO, USA). After incubation, the enzyme was inactivated with a culture medium: DMEM High Glucose (#L0102, Biowest, Nuaillé, France) with 20% FBS (#S181H, Biowest, Nuaillé, France) and 1% penicillin-streptomycin (#P4333, Sigma-Aldrich, St. Louis, MO, USA). Cells were transferred to 100 mm plates and incubated under standard culture conditions (37 °C, 5% CO_2_) for 72 h. Cells were then washed with DPBS (#L0615, Biowest, Nuaillé, France) to remove tissue debris and cultured in 20% FBS medium to achieve approximately 90% confluence. After the first passage, the cells were maintained in a complete culture medium: DMEM High Glucose (#L0102, Biowest, Nuaillé, France), 10% FBS (#S181H, Biowest, Nuaillé, France) and 1% penicillin-streptomycin (#P4333, Sigma-Aldrich, St. Louis, MO, USA).

HEK 293T cells (RRID:CVCL_0063, Cat# CRL-3216, ATCC, Manassas, VA, USA) were maintained in a complete culture medium: DMEM High Glucose (#L0102, Biowest, Nuaillé, France) with 10% FBS (#S181H, Biowest, Nuaillé, France) and 1% penicillin-streptomycin (#P4333, Sigma-Aldrich, St. Louis, MO, USA), under standard culture conditions. After reaching about 80–90% confluence, cells were passaged by trypsinization (#25-053-CI, Corning, Corning, NY, USA).

Generated iPSC and ESC (hES BG01V, RRID:CVCL_9727, Cat# SCRC-2002, ATCC, Manassas, VA, USA) were maintained in feeder-dependent culture under hypoxic conditions (37 °C, 5% CO_2_, 5% O_2_). Cells were cultured in plates coated with BD Matrigel™ Matrix, Basement Membrane, GFR (#354230, BD Biosciences, San Jose, CA, USA) and inactivated MEF cells (Cat#PMEF-CF, Millipore Merck KGaA, Darmstadt, Germany KGaA, Darmstadt, Germany) at 100% confluency, in iPSC medium: DMEM/F12 (#DF-042-B, Millipore Merck KGaA, Darmstadt, Germany KGaA, Darmstadt, Germany), 20% KSR (#10828028, Gibco, Thermo Fisher Scientific, Waltham, MA, USA), 1× NEAA (#M7145, Merck KGaA, Darmstadt, Germany), 1× β-mercaptoethanol (#ES-007-E, Merck KGaA, Darmstadt, Germany), 0.5% penicillin-streptomycin (#P4333, Sigma-Aldrich, St. Louis, MO, USA) and 10 ng/mL FGF (#PHG0264, Gibco, Thermo Fisher Scientific, Waltham, MA, USA). The medium was changed daily. Colonies were passaged after reaching about 70% confluence by incubation with 0.1% collagenase IV (#17104019, Gibco, Thermo Fisher Scientific, Waltham, MA, USA) in DMEM/F12 (#DF-042-B, Millipore Merck KGaA, Darmstadt, Germany KGaA, Darmstadt, Germany), for 2–5 min at 37 °C. After incubation, the enzyme was removed, the cells were gently washed with DPBS (#L0615, Biowest, Nuaillé, France), and a fresh iPSC medium was added. IPSC colonies were gently detached from the culture dish with a soft silicone scraper, carefully broken up in suspension into smaller aggregates with an automatic pipette, and transferred to a new Matrigel and MEF-coated culture vessel.

IPSC ND41658 (RRID:CVCL_Y836, Cat# ND41658, Coriell, Camden, NJ, USA) were cultured in feeder-free conditions in plates coated with BD Matrigel™ Matrix, Basement Membrane, GFR (#354230, BD Biosciences, San Jose, CA, USA). Cells were maintained in an Essential 8 Flex Medium Kit (#A2858501, Thermo Fisher Scientific, Waltham, MA, USA) under hypoxic conditions (37 °C, 5% O_2_, 5% CO_2_). The medium was changed every other day. Colonies were passaged after reaching about 60% confluence by incubation with a 0.48 mM EDTA (#E6511, Sigma-Aldrich, St. Louis, MO, USA) in DPBS (#L0615, Biowest, Nuaillé, France) for 3 min at 37 °C. After incubation, the solution was removed and residues inactivated with a culture medium. The colonies were gently detached from the plate using a soft silicone scraper, carefully broken up in suspension into smaller aggregates by pipetting, and transferred to new Matrigel-coated culture vessel.

### 2.3. Cloning Procedures

The pStemcca-TetO plasmid vector was constructed as previously described [15]. pLV-HK plasmid was a kind gift, generated in Didier Trono Lab, on the backbone of a pLV-tTRKRAB vector (RRID: Addgene_12249, Addgene, Watertown, MA, USA).

All generated vectors for the TRIM28 domains study were constructed on the 2nd generation lentiviral transfer plasmid pWPXL (RRID:Addgene_12257, Addgene, Watertown, MA, USA) backbone. Generation of 11 pWPXL vectors required complex multi-step cloning procedures. Detailed information on cloning steps is available from the Lead Contact. The hairpin targeted 5′-GCACTAGCTGTGAGGATAA-3′ of the endogenous *TRIM28* (457–476 nt). All exogenous variants of *TRIM28* contained a mutation introducing hairpin resistance 5′-GCACcAGtTGcGAaGAcAA-3′ (silent mutations marked with lowercase letters). Fragments of *TRIM28* phospho-mutants and phospho-mimetics were synthesized by GeneArt (Invitrogen, Carlsbad, CA, USA). Fragments of *TRIM28* structural mutants were obtained by directed mutagenesis (GeneArt Site-Directed Mutagenesis System, #A13282, Invitrogen, Carlsbad, CA, USA). All pWPXL- plasmids were sequenced (Genomed), and results were analyzed with Chromas 2.6.6 (RRID:SCR_000598, Technelysium Pty Ltd., https://technelysium.com.au/wp/chromas/, accessed on 27 June 2021). Mutagenesis and sequencing primers are listed in Table S2.

All plasmid vectors were transformed into *E. coli* JM109 (#P9751, Promega, Madison, WI, USA Research) and isolated with JETSTAR 2.0 Plasmid Maxiprep Kit (#220100, Gentaur, Kampenhout, Belgium).

### 2.4. Lentiviral Vectors Production, Purification, and Titration

Lentiviral vectors were produced by transient transfection of HEK 293T cells (ATCC, Manassas, VA, USA) by calcium phosphate precipitation. Briefly, HEK 293T (2.5 × 10^6^ cells) were seeded on 100 mm plates in a complete culture medium. The next day 20 μg of transfer plasmid, 15 μg of psPAX2 packaging plasmid (RRID:Addgene_12260, Addgene, Watertown, MA, USA), and 6 μg of pMD2G plasmid coding for virus envelope proteins G (RRID:Addgene_12259, Addgene, Watertown, MA, USA) were mixed with 50 μL 2.5 M CaCl_2_ (#C3881, Sigma-Aldrich, St. Louis, MO, USA), and sterile H_2_O to a volume of 500 μL. The mixture was added dropwise to 500 μL of 2X HBS buffer: 150 mM NaCl (#S3014, Sigma-Aldrich, St. Louis, MO, USA), 20 mM HEPES (#A3724, AppliChem, PanReac, Darmstadt, Germany) in H_2_O, and pH 7.05, with simultaneous aerating with an automatic pipettor. The volumes given are sufficient for one 100 mm plate. The transfection mixture was incubated for 5 min at RT and added to the cells. Cells were incubated for 6 h under standard culture conditions. Then, the medium was changed to 6 mL of fresh medium. The supernatant containing the virus particles was collected 48 h after transfection and centrifuged at 3000 rpm, 5 min, RT.

Virus-containing supernatant was purified and concentrated by ultracentrifugation in Ultra-Cone Polyallomer Centrifuge Tubes (Seton Scientific, Petaluma, CA, USA), in Sorvall Discovery 100S Ultracentrifuge (Kendro Laboratory Products, Asheville, NC, USA). Supernatants (20 mL) were carefully added dropwise on a 4 mL layer of 20% saccharose (#107651, Merck KGaA, Darmstadt, Germany) in distilled H_2_O. Tubes were centrifuged at 26,000 rpm, for 1.5 h at 4 °C. The supernatant was decanted, and the pellet (barely visible/invisible) was suspended in 2% BSA (#A9418, Sigma-Aldrich, St. Louis, MO, USA) in DPBS (#L0615, Biowest, Nuaillé, France) and incubated for 20 min at RT. The precipitate dissolved in the buffer was intensively pipetted, aliquoted, and stored at −80 °C.

Viral titers were determined by real-time PCR. HEK 293T cells were seeded at 20,000 cells/well of a 6-well plate in a complete culture medium. After 24 h, cells were transduced with each lentivirus produced, in volumes of 2, 5, and 10 μL, in duplicates. The flow cytometry-titered pWPXL lentiviral vector expressing EGFP was used as the reference. The medium was replaced 24 h after transduction with a fresh one, and the cells were cultured for another 5 days with the medium changed every other day. Cells were harvested by trypsinization (#25-053-CI, Corning, Corning, NY, USA), and DNA was isolated with Quick-DNA Miniprep (#D3025, Zymo Research, Irvine, CA, USA). All procedures were performed according to the manufacturer’s protocol. We used primers amplifying WPRE (present in integrating vector fragment) and GAPDH sequence. Primer sequences are listed in Table S2. Amplicons were detected with TaqMan hydrolysis probes (#04683633001, Roche, Basel, Switzerland). The number of infectious particles was calculated as described in the protocol [32].

### 2.5. Reprogramming of Human Fibroblasts towards iPSC

PHDF cells, in an early (2–3) passage, were seeded 10,000 cells/well of a 6-well plate in a complete culture medium. After 24 h and 48 h, cells were transduced with Stemcca-TetO lentiviral vector (50 IU/well). On day 7 after transduction, cells were passaged into 6-well plates, coated with BD Matrigel™ Matrix, Basement Membrane, GFR (#354230, BD Biosciences, San Jose, CA, USA), and MEF cells (#PMEF-CF, Millipore Merck KGaA, Darmstadt, Germany), approx. 4000/well. Cells were cultured in an iPSC medium as described in Cell culture, p. 3. The medium was changed every other day. Twenty-one days after transduction, the clusters of cells with stem cell-like morphology were manually transferred to freshly prepared wells of 6-well plates coated with Matrigel and MEF cells at 100% confluency. From this point, the iPSC medium was changed daily. After 2 passages, iPSC were transduced with LVE-HK lentiviral vector (hygromycin resistance), 10 IU/well, to silence the expression of exogenous reprogramming agents. IPSC were selected for 7 days with 50 μg/mL Hygromycin B (#H3274, Sigma-Aldrich, St. Louis, MO, USA).

### 2.6. Generation of TRIM28-Depleted or Mutated iPSC Populations

Silencing of *TRIM28* with siRNA in iPSC (obtained by reprogramming) was performed in 5 iPSC lines generated from different fibroblast donors, in duplicate. The results collected in this paper are representative results from the iPSC 26.6. iPSC were treated with a mix of two equimolar (50 nM) siRNA particles (Table S2) and Lipofectamine RNAiMAX Transfection Reagent (#13778-075, Invitrogen, Carlsbad, CA, USA), according to the manufacturer’s protocol. SiRNA BLOCK-iT™ Fluorescent Oligo (#2013, Invitrogen, Carlsbad, CA, USA) was used as a control in iPSC-CTRL. Transfection was performed every 72 h, for 21 days. TRIM28 expression was analyzed by real-time PCR and immunofluorescence staining.

Transduction of iPSC ND41658 with LV-WPXL carrying exogenous *TRIM28* variants was performed in suspension, in an Essential 8 Flex Medium Kit (#A2858501, Thermo Fisher Scientific, Waltham, MA, USA), with 10 µM Polybrene (#H9268, Sigma-Aldrich, St. Louis, MO, USA). Cells were transduced in 3 biological replicates (MOI = 20). IPSC were selected for 7 days with 0.3 mg/mL Puromycin (#P8833, Sigma-Aldrich, St. Louis, MO, USA).

### 2.7. Immunofluorescence Staining

Cells (seeded on 24-well plates) were washed 3 × 5 min with DPBS (#L0615, Biowest, Nuaillé, France) and fixed with a 4% formalin (#HT501128, Sigma-Aldrich, St. Louis, MO, USA) in DPBS, 10 min, at RT. Cells were washed 3 × 5 min with DPBS. The cell membrane was permeabilized (10 min, RT) with a 0.15% Triton X-100 (#T8787, Sigma-Aldrich, St. Louis, MO, USA) in DPBS and washed 3 × 5 min with DPBS. Cells were then incubated 30 min, RT with 1 mL blocking buffer: 1% BSA (#A9418, Sigma-Aldrich, St. Louis, MO, USA) and 0.1% Tween 20 (#P9416, Sigma-Aldrich, St. Louis, MO, USA) in DPBS. Cells were incubated with 200 μL primary antibodies in DPBS with 1% BSA, overnight, at 4 °C. Generated iPSC and hESC were stained every 7 days after transduction, for 3 weeks: anti-OCT3/4 (rabbit) 1:200; anti-NANOG 1:50; anti-SSEA4 1:50; anti-TRA-1-60 1:50; anti-TRA-1-81 1:50; anti-SSEA1 1:100. To siRNA-treated iPSC: anti-TRIM28 (rabbit) 1:50, anti-OCT3/4 (mouse) 1:200; anti-NANOG 1:50; anti-ATP5H 1:200; anti-PGK1 1:400; anti-PKM2 1:40; anti-HK1 1:250; anti-HK2 1:200. LV-treated ND41658 iPSC were stained after the 2nd, 6th, or 10th passage.: anti-TRIM28 (mouse) 1:200; anti-FLAG 1:800; anti-OCT3/4 (mouse) 1:200; anti-SOX2 1:200; anti-NANOG 1:50.

The next day, the cells were washed 3 × 5 min with DPBS and incubated 1 h in the dark, RT, with 200 μL fluorescently labeled secondary antibodies in DPBS with 1% BSA, in a ratio of 1:1000. After washing the cells three times with DPBS, the cell nuclei were stained with a DAPI (#32670, Sigma-Aldrich, St. Louis, MO, USA) in distilled water (1:10,000) for 5 min in the dark at RT. After washing the cells three times with DPBS, the cells were analyzed with Leica DMI3000B fluorescence microscope (Leica Microsystems, Wetzlar, Germany) and Leica Application Suite (RRID:SCR_016555, Leica Microsystems, Wetzlar, Germany). Primary and secondary antibodies are listed in Table S3.

### 2.8. cDNA Samples Preparation

Total RNA was isolated from 2 biological replicates (for siRNA-treated iPSC) or 3 biological replicates (for LV-treated ND41658 iPSC) using TRI Reagent (#T9424, Sigma-Aldrich, St. Louis, MO, USA). Reverse transcription was performed with an EvoScript Universal cDNA Master (#07912439001, Roche, Basel, Switzerland), according to the manufacturer’s protocol. One μg of total cellular RNA was used for each reaction. Obtained cDNA was diluted 10-fold in sterile DEPC water and used as an RT-PCR and real-time PCR template.

### 2.9. Real-Time PCR Quantification

Samples were amplified on a LightCycler^®^480 instrument (Roche, Basel, Switzerland). The gene expression level was quantified with 2^−ΔΔCT^ method, relative to the control sample. Primer sequences are listed in Table S2.

siRNA-treated iPSC were analyzed every 7 days after the first siRNA transfection for 3 weeks. Each reaction contained 2 μL cDNA, 1× LightCycler 480 Probes Master (#04887301001, Roche, Basel, Switzerland), 100 nM hydrolysis probes (Universal Probe Library), and 200 nM of primers for *TRIM28*, *OCT3/4*, *NANOG*, *CDX2*, *MSX1*, *FSP1*, *PAX6*, *SOX1*, *GAPDH*. Quantitative values for individual samples were normalized to *GAPDH*. Statistical results were calculated in GraphPad Prism6 (RRID:SCR_002798, GraphPad Software), using t-student and ANOVA tests.

LV-treated ND41658 iPSC were analyzed 6 or 10 passages after transduction. Each reaction contained 2 μL of cDNA template, 1× SYBR Green I Master Mix (#04707516001, Roche, Basel, Switzerland), and 200 nM of primers for *NANOG*, *LIN28A*, *BMP7*, *FGFR*, *PI3K*, *CDH1*, *ID1*, *TWIST2*, *PTCH1*, *SMO*, *NOTCH1*, *MAML1*, *WNT1*, *EPCAM*, *DKK1*, *DNMT3A*, *DNMT3B*, *TERT*, *EZH2*, *Nestin*, *TUJ*, *MAP2*, *GFAP*, *SMA*, *Brachyury*, *GATA4*, *GATA6*, *SOX17*, *B2M*, *HPRT1*, and *RPLP0*. The specificity of the amplified PCR product was assessed by melting curve analysis. Differentiated iPSC consisted of many types of cells, so quantitative determined values for individual samples were normalized to three reference genes: *B2M*, *HPRT1*, and *RPLP0*. Statistical results were calculated in GraphPad Prism6 (RRID:SCR_002798, GraphPad Software). One-way ANOVA variance analysis with a post-hoc Dunnett’s test was conducted.

### 2.10. Flow Cytometry

Cells were stained in 3 biological replicates, 10 passages after lentiviral transduction. Cell pellets were suspended in 100 μL DPBS (#L0615, Biowest, Nuaillé, France) with 1% BSA (#A9418, Sigma-Aldrich, St. Louis, MO, USA) and a primary antibody. The antibodies are listed in Table S3. Antibodies were administered in the following proportions: anti SSEA-4 1:50; anti TRA-1-60 1:50; anti TRA-1-81 1:50; anti IgG 1:50; anti IgM 1:50. Cells were incubated for 30 min at 4 °C and then washed 3 times with DPBS. Cell pellets were suspended in 100 μL 1% BSA in DPBS with biotin-conjugated secondary antibodies and incubated for 30 min at RT (IgG and IgM, 1:200). Cells were washed 3 times with DPBS, and pellets were suspended in 100 μL 1% BSA in DPBS and streptavidin APC conjugate (#17-4317-82, eBioscience, San Diego, CA, USA) and incubated at RT in the dark for 15 min. Cells were washed 3 times with DPBS, and pellets were suspended in 150 μL of 1× BD CellFIX fixing agent (#340181, BD Biosciences, San Jose, CA, USA). Cells were counted on a Becton Dickinson flow cytometer (BD Biosciences, San Jose, CA, USA FACSAria). The results were analyzed with the FlowJo_V10 (RRID:SCR_008520, FLOWJO, LLC Data Analysis Software).

### 2.11. RT-PCR

RT-PCRs were performed in 3 technical replicates with 2 μL of cDNA template, 500 nM primers (for *REX1*, *NODAL*, *DNMT3B*, *OCT3/4*, *GABRB3*, *NANOG*, Stemcca-TetO transgene or *GAPDH*), and ReadyMix™ Taq PCR Reaction Mix (#P4600, Sigma-Aldrich, St. Louis, MO, USA) according to the manufacturer’s protocols. Primer sequences are listed in Table S2.

### 2.12. Proliferation Assay

The proliferation ratio was determined in 3 biological replicates, with Cell Proliferation ELISA, BrdU, and colorimetric (#11 647 229 001, Roche, Basel, Switzerland), according to the manufacturer’s protocol. Briefly, 5000 cells were seeded in a 96-well plate in 100 µL/well and incubated at hypoxic conditions at 37 °C for 48 h. BrdU labeling was performed for 2 h. Cells were incubated with the anti-BrdU solution for 90 min and then with a substrate solution for 10 min. The absorbance was measured at 370 nm and calculated by subtracting the blank control absorbance value. Statistical results were calculated in GraphPad Prism6 (RRID:SCR_002798, GraphPad Software). One-way ANOVA variance analysis with a post-hoc Dunnett’s test was conducted.

### 2.13. Spontaneous In Vitro Differentiation Potential Assessment (Embryoid Bodies Formation)

Analysis was performed on 3 biological replicates of the iPSC 6 passages after lentiviral transduction. All variants of iPSC were harvested and washed twice with DPBS to remove Matrigel residues. Cells were then counted and seeded at 5000 cells/well on a 96 well, non-adherent, U-shaped plate in Essential 6™ Medium (#A1516401, Gibco, Thermo Fisher Scientific, Waltham, MA, USA) in standard culture conditions. Images and videos were taken with the Incucyte SX1 Live-Cell Analysis System (#4788, Sartorius, Göttingen, Germany), and the spheres’ area was calculated with ImageJ (RRID:SCR_003070). Statistical results were calculated in GraphPad Prism6 (RRID:SCR_002798, GraphPad Software) with a Kruskal–Wallis test followed by a Dunns’ test.

### 2.14. Western Blot

Cells were washed with DPBS (#L0615, Biowest, Nuaillé, France) and lysed in 200 μL of RIPA buffer (#J63306, Alfa Aesar, Ward Hill, MA, USA) at 4 °C, for 30 min. Cell lysates were centrifuged at 13,000 rpm at 4 °C for 30 min, and the supernatant was collected. Protein concentration was determined by BCA reaction with a Pierce™ BCA Protein Assay Kit (#23225, Thermo Fisher Scientific, Waltham, MA, USA), according to the manufacturer’s protocol. Further, 10 μg of protein was mixed with Laemmli Sample Buffer (#1610747, Bio-Rad Laboratories, Hercules, CA, USA) and RIPA to a final volume of 15 uL, and denatured at 95 °C, for 5 min. Electrophoresis was run in Tris/Glycine/SDS buffer (#1610772, Bio-Rad Laboratories, Hercules, CA, USA) on a Mini-PROTEAN TGX Precast Gel (#4561086, Bio-Rad Laboratories, Hercules, CA, USA), with Precision Plus Protein Kaleidoscope Pre-stained Protein Standards (#1610375, Bio-Rad Laboratories, Hercules, CA, USA). Proteins were transferred on a PVDF membrane, Trans-Blot Turbo Transfer Pack (#1704156, Bio-Rad Laboratories, Hercules, CA, USA). The membrane was blocked for 30 min in 5% milk in TBST buffer: 0.01 M TRIS (#T1503, Sigma-Aldrich, St. Louis, MO, USA), 0.15 M NaCl (#S3014, Sigma-Aldrich, St. Louis, MO, USA) and 0.1% Tween 20 (#P9416, Sigma-Aldrich, St. Louis, MO, USA). Membrane fragments were incubated with primary antibodies in 5 mL 5% milk in TBST buffer, at 4 °C, overnight. Membranes were washed 3 times with 10 mL TBST buffer, for 10 min, and incubated with secondary HRP-conjugated antibodies in 5 mL 5% milk in TBST buffer, followed by triple washing with 10 mL TBST buffer. Antibodies were visualized with a WesternBright Quantum HRP substrate (#K-12042, Advansta, San Jose, CA, USA). Antibodies are listed in Table S3.

### 2.15. Teratoma Formation

iPSC were harvested with 0.1% collagenase IV (#17104019, Gibco, Thermo Fisher Scientific, Waltham, MA, USA), and 2 × 10^6^ of cells were resuspended in 50 μL iPSC medium. Before injection, 50 μL of BD Matrigel™ Matrix Basement Membrane GFR (#354230, BD Biosciences, San Jose, CA, USA) was added to the cell suspension at 4 °C, and the mixture was injected subcutaneously into the lower flank of immunodeficient NOD SCID mice NOD.CB17-*Prkdc^scid^*/NCrCrl (Charles River Laboratories, Wilmington, MA, USA). All animal experiments were performed following institutional guidelines. After 7–9 weeks, tumors were resected, measured, and subjected to RNA isolation and immunohistochemical staining. Paraffin sections of formalin-fixed teratomas were stained with hematoxylin and eosin (H + E) and antibodies specific for markers of three germ layers: endoderm cytokeratins, ectoderm GFAP, and mesoderm desmin. Analysis was performed in the Department of Tumor Pathology, Greater Poland Cancer Centre in Poznań.

### 2.16. Karyotyping of Generated iPSC Lines

Karyotype analyses were performed by the Cytogenetic Laboratory, Cancer Centre-Maria Sklodowska–Curie Institute in Warsaw, according to the standard protocol for G-banding.

### 2.17. Bisulfite Sequencing for Promoter Methylation Analysis

DNA from 8 iPSC clones and 4 PHDF lines was isolated and bisulfite-treated as described [8]. DNA from 8 iPSC clones and 4 PHDF cell lines was isolated with a Quick-DNA Miniprep Kit (#D3025, Zymo Research, Irvine, CA, USA). Then, 1.5 µg DNA was bisulfite converted with EZ DNA Methylation Kit (#D5001, Zymo Research, Irvine, CA, USA), according to the manufacturer’s protocol. *OCT3/4* and *NANOG* promoter was amplified with 500 nM specific primers (listed in Table S2) and the GoTaq Green Master Mix (#M7122, Promega, Madison, WI, USA). PCR was performed with the following thermal profile: 95 °C/2 min, 42 cycles of 95 °C/30 s + 61 °C/30 s + 72 °C/30 s, and a final extension at 72 °C for 7 min. Amplified fragments of *OCT3/4* and *NANOG* promoters were ligated to pGEM-T Easy vector (#A1360, Promega, Madison, WI, USA) with a LigaFast Rapid DNA Ligation System (#M8221, Promega, Madison, WI, USA) and cloned into *E. coli* DH5a (#T3007, Zymo Research, Irvine, CA, USA). The plasmids from individual clones were purified with a Zyppy™ Plasmid Miniprep Kit (#D4036, Zymo Research, Irvine, CA, USA), sequenced (Genomed, Warsaw, Poland), and analyzed with the BISMA application (RRID:SCR_000688, Jacobs University Bremen; Germany) [33]. The percentage of methylation (MtI) was calculated according to the formula: MtI% = Cm/(Cm + Cnm).

### 2.18. Proteomic Profiling–Reverse Phase Protein Array (RPPA)

Analysis was performed on 1 biological replicate. Pellets of PHDF cells and derived iPSC were washed with DPBS (#L0615, Biowest, Nuaillé, France) and lysed in 100 μL of RIPA buffer (#J63306, Alfa Aesar, Ward Hill, MA, USA). Cell lysates were centrifuged at 13,000 rpm, at 4 °C for 30 min, and supernatant was collected. Protein concentration was determined by BCA reaction with a Pierce™ BCA Protein Assay Kit (#23225, Thermo Fisher Scientific, Waltham, MA, USA), according to the manufacturer’s protocol. Each sample (40 μL) was mixed with 4X SDS sample buffer: 40% Glycerol (#A1123, AppliChem, PanReac, Darmstadt, Germany), 8% SDS (#L4509, Sigma-Aldrich, St. Louis, MO, USA), 0.25 M Tris-HCl (#10812846001, Roche, Basel, Switzerland), pH 6.8, and 10% β-mercaptoethanol (#ES-007-E, Merck KGaA, Darmstadt, Germany), and boiled for 5 min. Samples were stored in −80 °C. Samples were analyzed in the RPPA Core Facility, The University of Texas, MD Anderson Cancer Center (Houston, TX, USA). Each sample was diluted in five 2-fold serial dilutions in 1% SDS lysis buffer. Serially diluted lysates were arrayed on nitrocellulose-coated ONCYTE^®^ Film slides (Grace Bio-Labs, Bend, OR, USA) by Aushon 2470 Arrayer (Aushon BioSystems, Billerica, MA, USA) in 11 × 11 format. Each slide was probed with a validated primary antibody plus a biotin-conjugated secondary antibody. A total of 305 unique antibodies were used. The signal obtained was amplified using a Dako Cytomation–catalyzed system (Agilent Technologies, Santa Clara, CA, USA) and visualized by DAB colorimetric reaction. The slides were scanned, analyzed, and quantified using MicroVigene (RRID:SCR_002820, VigeneTech Inc., Carlisle, MA, USA) software to generate spot intensity. Each dilution curve was fitted with a logistic model SuperCurve Fitting (Department of Bioinformatics and Computational Biology in MD Anderson Cancer Center, Houston, TX, USA) utilizing the R environment (RRID:SCR_001905, CRAN family, http://www.r-project.org/, accessed on 27 June 2021). The fitted curve was plotted with the signal intensities on the y-axis and the log2-concentration of proteins on the x-axis. The protein concentrations of each set of slides were then normalized by median polish, corrected across samples by the linear expression values. A correction was performed using the median expression level of all antibody experiments to calculate a loading correction factor for each sample.

### 2.19. Principal Component Analysis

Principal components were calculated using the ClustVis web tool [34] (RRID:SCR_017133, University of Tartu, Tartu, Estonia). The imputation and Singular Value Decomposition (SVD) were performed iteratively until estimates of missing values converge was performed. As an input, linear normalized RPPA data were used for all tested samples. The first two principal components (PC1, PC2) were plotted.

### 2.20. Differential Expression Analysis

Proteins from RPPA data were filtered based on the adjusted *p*-value < 0.05 and presented in volcano plots. All significantly differentially expressed proteins were clustered with the Morpheus tool [35] (RRID:SCR_014975, Dresden University of Technology, Dresden, Germany) and visualized as heatmaps. The distance was calculated with one minus Pearson’s correlation coefficient metric.

### 2.21. Gene Set Enrichment Analysis (GSEA)

GSEA (http://www.broad.mit.edu/gsea/index.html access date: 26 March 2020) was used to detect the coordinated expression of a priori defined groups of genes within the tested samples. Gene sets are available from the Molecular Signatures Database (MSigDB, RRID:SCR_016863, Broad Institute, Cambridge, MA, USA, http://software.broadinstitute.org/gsea/msigdb/index.jsp access date: 26 March 2020). Briefly, GSEA generated an enrichment score (ES) reflective of the degree to which a gene set is overrepresented at the extremes (top or bottom) of the entire list of RPPA data. Genes are ranked according to expression difference (signal/noise ratio) between the tested group of samples: PHDF-WT/PHDF-CTRL and iPSC-WT/iPSC-CTRL, where CTRL cells are treated with a control siRNA. The ES calculation and estimation of the *p*-value, together with the normalized enrichment score (NES) and FDR calculations, have been previously described in detail [36]. A total of 305 markers (previously ranked based on their log2FC between analyzed groups) were imported for GSEA. The GSEA was run according to the default parameters: each probe set was collapsed into a single gene vector (identified by its HUGO gene symbol), permutation number = 1000; permutation type = “gene-sets.” The FDR was used to correct for multiple comparisons and gene set sizes.

### 2.22. Over-Representation Enrichment Analysis (ORA)

The ORA [37] was performed with a WEB-based Gene SeT AnaLysis Toolkit (WebGestalt; http://www.webgestalt.org/ access date: 28 March 2020) with the “pathway” database. Protein names were transferred into gene symbols, and the reference gene list was set at “genome protein-coding”. Upregulated and down-regulated markers were considered separately.

## 3. Results

### 3.1. Generating Human iPSC with Doxycycline-Inducible System Results in Repression of Transgene Expression in Established Clones

IPSC were generated by reprogramming primary human dermal fibroblasts (PHDFs) with Yamanaka factors: *OCT3/4*, *SOX2*, *KLF4*, and *c-MYC* (OSKM) [38], delivered in Stemcca-TetO lentivirus, under the control of the doxycycline-inducible system (Figure S2A). PHDF cell lines were established from the healthy skin margins surrounding breast cancers excised during mastectomy. Application of the inducible system allowed to switch off OSKM expression in obtained iPSC colonies by transducing them on day 21 with LV-HK lentivirus. LV-HK vector carried the expression of the KRAB tet-repression domain, which, in the absence of doxycycline, inhibits EF-1α promoter (Figure S2A). Re-induction of transgene expression would require the presence of doxycycline in culture media.

Obtained iPSC displayed typical [38] round colony morphology with a clear, regular peripheral outline (Figure S2B). Colonies expressed intra- and extra-cellular pluripotency markers (positive for OCT3/4, NANOG, SSEA-4, TRA-1-60, and TRA-1-81, and negative to differentiation marker SSEA-1), comparable to the expression profile of hESC (Figure S2C). The CpG methylation status in the promoter regions of *OCT3/4* and *NANOG* was evaluated by bisulfite sequencing. The analyzed promoters were unmethylated in iPSC compared to parental PHDF cells, indicating the activity of these promoters in iPSC (Figure S2D). Moreover, we confirmed a similar expression profile of pluripotency markers (REX1, NODAL, DNMT3B, OCT3/4, GABRB3, NANOG) in hESC and obtained iPSC (Figure S2E). Chromosomal G-band analysis showed normal karyotypes with no chromosomal aberrations in generated lines (Figure S2F). Immunohistochemical and H + E staining of iPSC-derived teratoma sections proved iPSC potential to differentiate into ecto-, endo- and mesoderm (Figure S2G). We also confirmed no transgene expression from the integrated Stemcca-TetO vector in teratomas (Figure S2H).

### 3.2. Silencing of Endogenous TRIM28 Induces Downregulation of Pluripotency Markers and Differentiation of Human iPSC

**Figure 1:**
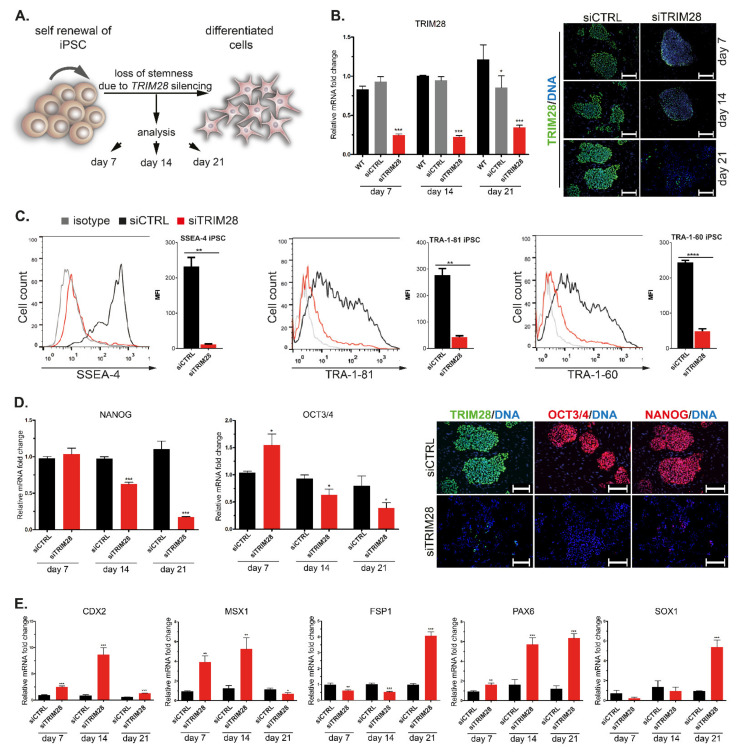
Silencing of endogenous *TRIM28* induces downregulation of pluripotency markers and differentiation of human iPSC. (**A**) Endogenous *TRIM28* was silenced in human iPSC with siRNA. Stemness and differentiation status were analyzed every 7 days for 3 weeks. (**B**) Silencing of TRIM28 at transcriptional and protein level confirmed by qRT-PCR (left) and immunofluorescence staining (right; green—TRIM28, blue—DAPI). Scale bar: 100 μm. Representative images are shown in the figures. (**C**) Flow cytometry analysis revealed decreased expression of extracellular pluripotency markers SSEA-4, TRA-1-60, and TRA-1-81 in siTRIM28 iPSC (red) vs. CTRL iPSC (black). Isotype control is marked in gray. (**D**) The progressive loss of intracellular pluripotency markers (NANOG, OCT3/4) expression in siTRIM28 iPSC confirmed with qRT-PCR (left) and immunofluorescence staining (right). Cells were stained at day 21. Scale bar: 100 μm. (**E**) qRT-PCR of differentiation-associated markers shows their upregulation during spontaneous differentiation of siTRIM28 iPSC. (**B**–**E**) Graphs represent mean (SD), *n* = 3. Statistical analysis was performed with t-student and ANOVA tests; *p* ≤ 0.05 (<0.0001 ****; 0.0001–0.001 ***; 0.001–0.01 **; 0.01–0.05 *; ≥0.05 not significant).

To evaluate the role of TRIM28 in stemness, we silenced endogenous *TRIM28* expression with small interfering RNA (siRNA) in two generated iPSC lines. IPSC treated with siRNA with no target sequence served as a control (siCTRL) of the experiment. Upon *TRIM28* silencing, the stemness and differentiation status was examined every 7 days for 3 weeks (Figure 1A). The silencing efficiency was confirmed on transcriptional and protein level by qRT-PCR and immunofluorescence staining (Figure 1B). The significant downregulation of *TRIM28* in the CTRL sample at day 21 might result from that control siRNA, and transfection reagents can influence mRNA and protein levels [39]. Upon *TRIM28* silencing, we observed decreased expression of extracellular pluripotency markers, SSEA-4, TRA-1-60, TRA-1-81 (Figure 1C), and progressive loss of intracellular pluripotency markers OCT3/4 and NANOG (Figure 1D). Finally, differentiation-associated markers (*CDX2*, *MSX1*, *FSP1*, *PAX6*, *SOX1*) were upregulated in siTRIM28 cells on the transcript level (Figure 1E). Our data indicate that *TRIM28* knock-down facilitates differentiation of iPSC.

### 3.3. iPSC with Silenced TRIM28 Display Metabolic Changes, and Their Proteomic Profile Differs from the Control iPSC

**Figure 2:**
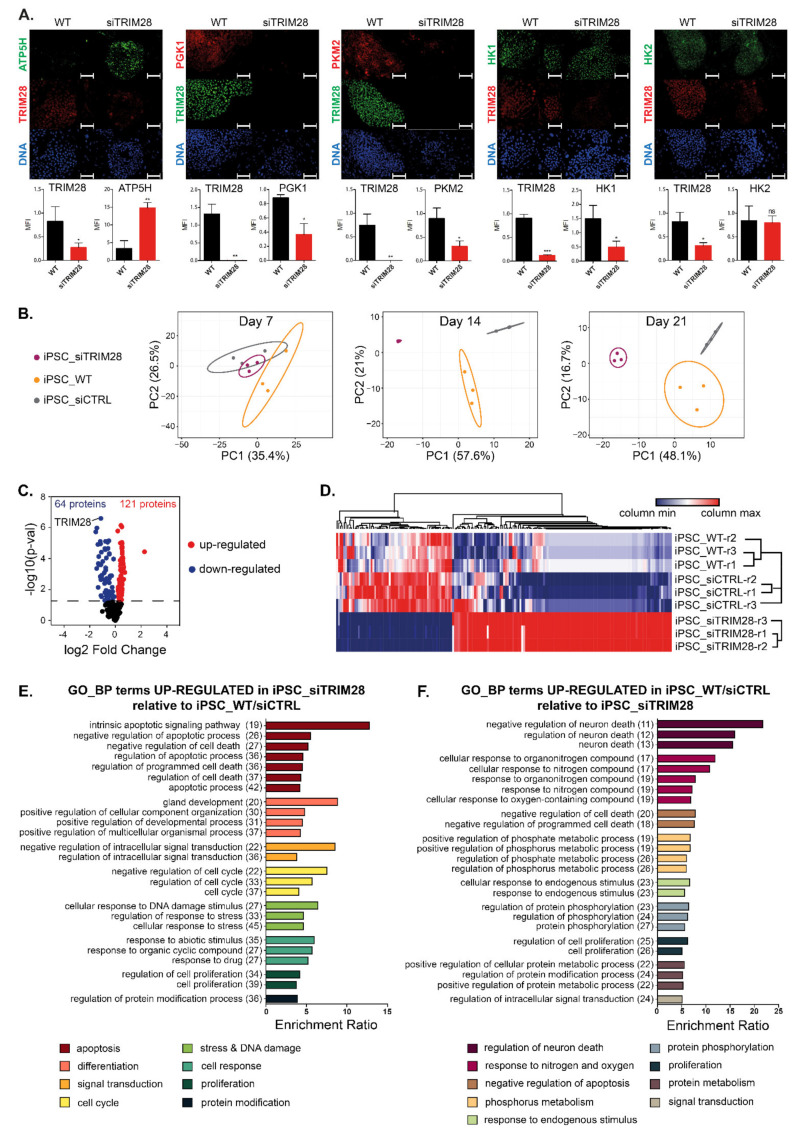
IPSC with silenced *TRIM28* display metabolic changes and their proteomic profile differs significantly from the control iPSC. (**A**) Representative images of immunofluorescence staining showing metabolic changes from glycolysis (PGK1, PKM2—red; HK1, HK2—green) to oxidative phosphorylation (ATP5H—green) in differentiating iPSC-siTRIM28 (TRIM28—green/red; DAPI—blue). Scale bar 100 μm. Graphs represent the quantitative analysis of MFI (SD), *n* = 3. Cells were analyzed on day 14 upon *TRIM28* silencing. (**B**) Principal Component Analysis (PCA) of RPPA data from 3 timepoints upon iPSC differentiation, *n* = 1. Numbers in parentheses represent the percentage of total variance explained by the first and the second PC. Prediction ellipses are such that with a probability 0.95, a new observation from the same group will fall inside the ellipse. Dots represent samples: purple—iPSC-siTRIM28, yellow—iPSC-WT, grey—iPSC-CTRL. (**C**) Protein level changes between iPSC-siTRIM28 cells and reference iPSC (WT and CTRL). The number of significantly downregulated (blue) or upregulated (red) proteins, with *p*-val < 0.05, is presented on the volcano plot, *n* = 1. (**D**) Heatmap of 185 differentially expressed markers in WT/CTRL and siTRIM28 cells, based on RPPA data (*p*-val < 0.05). The relative protein level is presented as z-score. Blue—minimal value, red—maximal value in each column, *n* = 1. (**E**,**F**) Gene Ontology Biological Processes significantly enriched in the list of upregulated markers in iPSC-siTRIM28 (**E**) and reference iPSC (**F**), as determined with Overrepresentation Enrichment Analysis (ORA) using WebGestalt on-line tool. Numbers in parentheses indicate the number of markers in individual processes. Top 25 terms with FDR < 0.05% are presented.

Two weeks after silencing *TRIM28*, we evaluated metabolic changes by immunofluorescence staining (Figure 2A). During reprogramming of somatic cells into iPSC, the metabolic profile shifts from oxidative phosphorylation (OXPHOS) to glycolysis [40,41,42]. Upon cellular differentiation, the metabolic profile shifts back to OXPHOS, and mitochondrial activity is restored. Silencing of *TRIM28* resulted in high upregulation of mitochondrial ATP synthase subunit D (ATP5PD), which indicates OXPHOS metabolism in differentiated cells. Evident downregulation of glycolysis-related markers, including phosphoglycerate kinase (PGK), pyruvate kinase (PKM2), and hexokinase 1 (HK1), compared to wild type (WT) cells, also confirmed inhibition of glycolysis processes as a result of differentiation. The expression of HK2 did not change significantly upon *TRIM28* silencing.

iPSC with silenced *TRIM28* were subjected to RPPA analysis, and their proteomic profile was compared with WT and siCTRL iPSC. Raw data are openly available in GEO at www.ncbi.nlm.nih.gov/geo access date: 3 July 2020, reference number: GSE153726. Principal Component Analysis (PCA) showed that the samples from each group clustered together, and the groups were clearly segregated (Figure 2B). We determined 185 differentially expressed proteins, of which 64 were significantly downregulated and 121 upregulated between iPSC siTRIM28 and reference iPSC (WT and siCTRL) (Figure 2C). Differentially expressed proteins showed clustering of markers from iPSC_WT, iPSC_siCTRL, and iPSC_siTRIM28 samples (Figure 2D). Pathway enrichment analysis using Gene Ontology datasets showed significant upregulation of pathways related to apoptosis, differentiation, cellular response to DNA damage stimulus, and cell cycle regulation in iPSC-siTRIM28, relative to reference iPSC (Figure 2E). In contrast, reference iPSC demonstrated enrichment of the processes involved in the cellular response to organonitrogen and nitrogen compounds and processes related to the regulation of phosphorylation and cell proliferation (Figure 2F). The list of markers assigned to individual processes is presented in Figure S3. Among the markers upregulated upon *TRIM28* silencing, we found some tumor suppressor genes, e.g., MSH2, CHEK2, ANXA, or CAV1 (Figure S3A). One of the downregulated markers in iPSC-siTRIM28 (upregulated in reference iPSC) was TRIM28, and a few protooncogenes, e.g., EIF4E, BRAF, ARAF (Figure S3B). These results indicate the role of TRIM28 in the regulation of several signaling pathways implicated in maintaining self-renewal and stemness of iPSC, and probably of highly dedifferentiated metastatic tumor cells as well.

### 3.4. Selection of Mutation Sites Impairing the Function of TRIM28 Protein Domains

**Figure 3:**
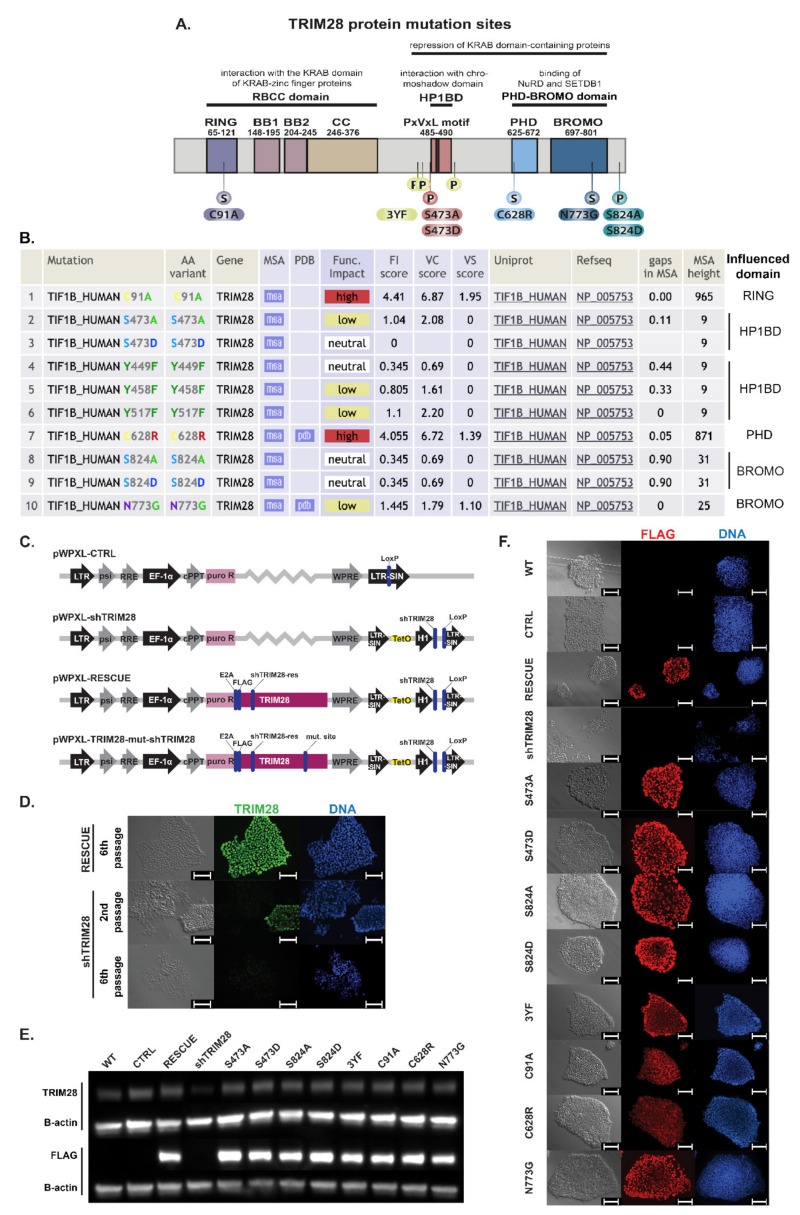
The constructed lentiviral system enables efficient transgene expression and silencing of endogenous *TRIM28*. (**A**) Localization of structural (S) or phosphorylation (*p*) mutations chosen to affect the function of each TRIM28 domain. (**B**) Prediction of the functional impact of selected mutations on TRIM28 activity performed with Mutation Assessor algorithms. Table source: http://mutationassessor.org/ access date: 17 February 2019. MSA—annotated in multiple sequence alignment (MSA) browser. PDB—annotated in 3D structure browser. FI score—functional impact combined score. VC score—variant conservation score. VS score—variant specificity score. Gaps in MSA—portion of gaps in variant position in MSA, MSA height—number of diverse sequences in multiple sequence alignment. (**C**) Lentiviral system generated to silence endogenous *TRIM28* by shRNA (shTRIM28) and express exogenous, shRNA-resistant (shTRIM-res), and FLAG-tagged *TRIM28* sequences. (**D**) Silencing of endogenous TRIM28 and shRNA specificity to endogenous *TRIM28* sequence evaluated by immunofluorescence staining (green—TRIM28, blue—DAPI). Scale bar: 100 μm. (**E**) Silencing of endogenous TRIM28 and expression of exogenous FLAG-tagged proteins confirmed by Western Blot. (**F**) Efficient transgene expression assessed by immunofluorescence staining against FLAG-tag attached to N-terminus of exogenous TRIM28 protein (red—FLAG, blue—DAPI). Scale bar: 100 μm.

To determine the TRIM28-dependent mechanisms responsible for the self-renewal and pluripotency maintenance, we selected eight different mutation sites within *TRIM28*. Mutations impaired the functions of its particular domains by their effect on phosphorylation of the domain structure (Figure 3A,B). All mutations introduced into the *TRIM28* sequence and predicted effects on protein activity are summarized in Table 1.

Five of the *TRIM28* mutation sites were chosen based on the literature reports related to the key amino acids of the TRIM28 protein undergoing phosphorylation that affect particular domains’ activity. Phosphorylation on Ser474, which is located directly before HP1BD, lowers the ability to bind HP1 protein and inhibits TRIM28 transcription repressor activity [31,43]. TRIM28 phosphorylation on three tyrosines, Y449F/Y458F/Y517F (3YF), flanking HP1BD, also reduces the HP1 binding ability, preventing silencing of gene expression [19]. Phosphorylation on Ser824 affects the activity of BROMO by decreasing TRIM28 sumoylation and is associated with relaxed chromatin. It blocks the differentiation of mouse pluripotent cells and induces the expression of *SOX2* and *NANOG* [18,44].

**Table 1:** Selected *TRIM28* mutation sites with predicted effects on protein activity.

Mutation	Domain	Predicted Influence on TRIM28 Activity	Reference
C91A (structural mutant)	RING	Destabilization of RING domain structure	[28,45,46,47,48]
		Inhibition of interaction with KRAB domain of KRAB-ZNFs	
		Impairment of E3 ubiquitin ligase function	
		Restriction of TRIM28 transcription co-repressor function	
S473A (phospho-mutant)	HP1BD	Inhibition of TRIM28 Ser473 phosphorylation	[31,43]
		HP1BD interaction with HP1	
		TRIM28 functions as transcription co-repressor	
S473D (phospho-mimetic)	HP1BD	Imitation of permanent TRIM28 Ser473 phosphorylation	[31,43]
		Inhibition of HP1 binding by HP1BD	
		Restriction of TRIM28 transcription co-repressor function	
3YF (phospho-mutant)	HP1BD	Inhibition of triple tyrosine phosphorylation	[19]
		HP1BD interaction with HP1	
		TRIM28 functions as a transcription repressor	
C628R (structural mutant)	PHD	Destabilization of PHD domain structure	[30,49]
		Impairment of E3 SUMO ligase function	
		Inhibition of NuRD complex and SETDB1 methyltransferase binding	
		Restriction of TRIM28 transcription repressor function	
N773G (structural mutant)	BROMO	Destabilization of bromodomain structure	-
		Low influence on TRIM28 activity	
S824A (phospho-mutant)	BROMO	Inhibition of TRIM28 Ser824 phosphorylation	[18,44]
		Increased TRIM28 BROMO sumoylation	
		Induction of differentiation	
S824D (phospho-mimetic)	BROMO	Imitation of permanent TRIM28 Ser824 phosphorylation	[18,44]
		Decreased TRIM28 BROMO sumoylation	
		Inhibition of spontaneous differentiation	
		Induction of SOX2 and NANOG expression	

Structural mutants were selected using Mutation Assessor algorithms (RRID:SCR_005762, Computational Biology Center, Memorial Sloan Kettering Cancer Center) [50]. The algorithm analyzes protein family multiple sequence alignments (MSA) of homologous sequences, exploits sequence homologs 3D structures, and generates conservation scores to predict functional specificity. The functional impact score of mutation is calculated based on the evolutionary conservation in a protein family and, separately, in every subfamily [50].

The C91A mutation in the RING domain results in the abolition of E3 ubiquitin ligase function and inhibition of binding to transcription factors containing the KRAB domain, leading to the loss of the transcription repressor function [45,46,47,48]. Structural C628R mutation in PHD inhibits its endogenous E3 SUMO ligase function and hindrance of BROMO sumoylation [28]. It impedes the interaction with the NuRD complex and SETDB1 methyltransferase and restricts the function of TRIM28 as a transcription repressor [30,49]. The N773G structural mutation, located in BROMO, was selected as a structural control mutation that has a low impact on the impairment of protein function.

### 3.5. Mutations in RING and PHD Domains Are Classified as Mutations with a High Impact on TRIM28 Function

Predictive algorithms indicated C91A and C628R to be mutations having a high impact on the function of the TRIM28 protein (Figure 3B). Many previous reports demonstrated common TRIM28 phosphorylation sites (S473, 3YF, S824) as crucial for TRIM28 function. Experiments were performed on mouse ESC [18], HEK-293 cells [31,43,44], breast cancer cell line MCF-7 [44], or cervical cancer cell line HeLa [19,31,43]. In contrast, our predictive analysis indicated that phosphorylation site mutations have a low or neutral functional impact.

### 3.6. Constructed Lentiviral System Enables Efficient Transgene Expression and Silencing of Endogenous TRIM28

To obtain more homogenous populations and reduce the factors that might influence the subtle phenotype changes, we decided to perform further experiments on commercial feeder-free human iPSC line ND41658*H (NINDS Human Genetics DNA and Cell Line Repository, Coriell, Camden, NJ, USA). We also wanted to evaluate whether previously observed differentiation due to *TRIM28* silencing will be observed on other iPSC lines.

To generate populations expressing exogenous *TRIM28* or its mutated variants, iPSC were transduced with lentiviral vectors carrying mutated exogenous *TRIM28* sequence and shRNA sequence silencing endogenous *TRIM28* (mut-shTRIM28) (Figure 3C). We also generated the empty vector control population (CTRL) only with silenced endogenous *TRIM28* expression (shTRIM28) and population expressing exogenous non-mutated *TRIM28* and hairpin silencing endogenous TRIM28 (RESCUE). Exogenous mutated (S473A, S473D, 3YF, S824A, S824D, C91A, C628R, N773G) and non-mutated (RESCUE) *TRIM28* sequences were resistant to shRNA (shTRIM28-res) and were tagged at the N-terminus with FLAG sequence.

First, we evaluated the shRNA effect on endo- and exogenous TRIM28 expression by immunofluorescence staining (Figure 3D). At the initial stages (2nd passage), we observed some colonies expressing TRIM28 in cells transduced with the shTRIM28 vector, probably due to ongoing and not yet complete puromycin selection. After the 6th passage, we re-assessed *TRIM28* silencing, and this time TRIM28 expression was decreased in all studied colonies. The control RESCUE population expressed TRIM28 among all the colonies, confirming resistance of exogenous sequence to shRNA.

Analysis of anti-FLAG antibodies proved the functionality of applied lentiviral vectors. We detected transgene expression in all populations of iPSC with exogenous variants of *TRIM28*, but the intensity varied within transduced populations (Figure 3E) and cells in colonies (Figure 3F). Heterogenous transgene expression might result from transducing iPSC as small aggregates, not as a single cell suspension, as well as from non-clonal selection.

### 3.7. Mutations of RING and PHD Domains Influence Human iPSC Morphology and Pluripotency Markers Expression

**Figure 4:**
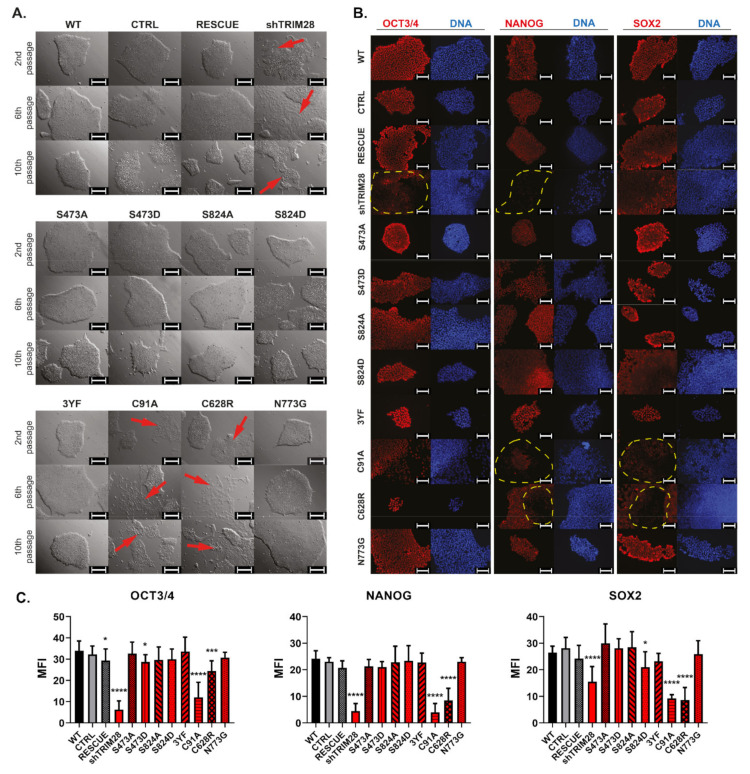
Mutations of RING and PHD domains influence iPSC morphology and pluripotency marker expression. (**A**) Morphology of iPSC colonies was visually inspected every day for 10 passages after LV transduction. Pictures show representative morphology after the 2nd, 6th, and 10th passage. Scale bar: 200 µm. (**B**,**C**) Loss of endogenous pluripotency markers expression in differentiating populations (shTRIM28, C91A, C628R) evaluated by immunofluorescence staining against OCT3/4, SOX2, and NANOG (red—pluripotency markers; blue—DAPI). Scale bar: 100 µm. Graphs represent the quantitative analysis of mean MFI (SD), *n* = 3. Statistical analysis performed with one-way ANOVA and a post-hoc Dunnett’s test; *p* ≤ 0.05 (<0.0001 ****; 0.0001–0.001 ***; 0.01–0.05 *; ≥0.05 not significant).

First, morphological changes appeared right after the 1st passage in the variant with the silenced endogenous *TRIM28* transcript (shTRIM28). After the 2nd passage, similar changes were observed in iPSC with mutations within RING (C91A) and within PHD (C628R) (Figure 4A). These populations demonstrated the loss of morphology typical for pluripotent cells [38]. Non-modified cell colonies (WT) and cells transduced with control vectors (CTRL and RESCUE) showed no changes in the characteristic uniform morphology. Among other modified variants of iPSC, no morphology changes indicating differentiation were noted. Occasionally, differentiating single cells were left among the undifferentiated colonies to not interfere with the differences arising between individual variant populations.

Silencing of endogenous *TRIM28* (shTRIM28) resulted in downregulation of OCT3/4 expression compared to other analyzed populations (Figure 4B). On the other hand, a downregulated level of NANOG was demonstrated in the shTRIM28 population and, to a smaller extent, in populations with mutated RING (C91A) or PHD domains (C628R). In differentiating populations (shTRIM28, C19A, and C628R), the SOX2 level was also reduced. Also, in some cells of the S824D population, SOX2 was decreased, but no other signs of cell differentiation were noted, and no changes in colony morphology after the 10th passage were found. The remaining phospho-mutants and phospho-mimetics did not affect the expression of the analyzed pluripotency markers.

### 3.8. Dysfunction of RING and PHD Domains Results in Decreased Proliferation and Inhibition of Embryoid Bodies Formation 

**Figure 5:**
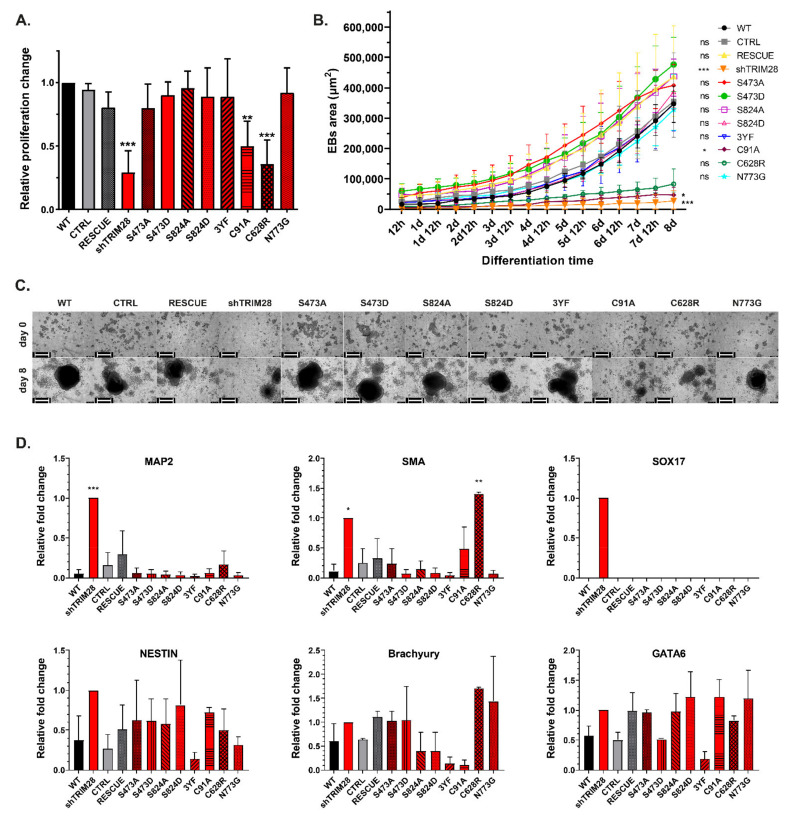
Dysfunction of RING and PHD domains results in decreased proliferation and inhibition of embryoid body formation. (**A**) Changes in iPSC proliferation calculated by spectrophotometry analysis of BrdU incorporation on the 6th passage after LV transduction (*n* = 3). (**B**) EBs area was calculated from images taken by Incucyte for 8 days after induction of EBs formation. Each bar represents the mean area in µm (SD), *n* = 3. Statistical analysis performed with Kruskal–Wallis test; *p* ≤ 0.05 (0.0001–0.001 ***; 0.001–0.01 **; 0.01–0.05 *; ≥ 0.05 not significant). (**C**) Example images taken by Incucyte during EBs formation (*n* = 3). Scale bar: 400 µm. See also Videos S1–S12. (**D**) QRT-PCR analysis of differentiation markers in iPSC populations 6 passages after LV transduction. Graphs represent markers related to ectoderm (Nestin, MAP2), mesoderm (SMA, Brachyury), endoderm (GATA6, SOX17). Each bar represents the mean mRNA expression level (SD), *n* = 3, SOX17: *n* = 1. If no amplification occurred, the expression level was calculated as 0. Statistical analysis performed with one-way ANOVA and a post-hoc Dunnett’s test; *p* ≤ 0.05 (0.0001–0.001 ***; 0.001–0.01 **; 0.01–0.05 *; ≥0.05 not significant).

We also evaluated the influence of *TRIM28* mutations on proliferation and in vitro spontaneous differentiation potential (Figure 5). As expected, differentiating populations (shTRIM28, C19A, and C628R) displayed a decreased proliferation compared to WT or CTRL/RESCUE iPSC (Figure 5A). However, analyzed phospho-mutants and phospho-mimetics did not significantly affect proliferation.

Differentiation potential was analyzed by EBs formation. EBs were formed by forced aggregation in a non-adherent 96-well plate and monitored with an Incucyte SX1 Live-Cell Analysis System (#4788, Sartorius, Göttingen, Germany). We observed smaller or no spheres derived from shTRIM28, C91A, and C628R compared to the rest of the populations (Figure 5B,C, Videos S1–S12). We assumed this effect was caused by differentiation induction after impairment of the RING and PHD function. Therefore we evaluated differentiation markers expression in the populations used to obtain EBs (Figure 5D). Unfortunately, due to late or no amplification in many samples, SD is very high and disabled drawing conclusions. However, we observed a clear trend in the expression of *MAP2* (ectoderm) in shTRIM28 and *SMA* (mesoderm) in shTRIM28 and C628R cells. MAP2 is a neuronal marker, yet its expression can be found in differentiating EBs, as soon as 16 days upon EBs formation, which might explain its presence in differentiating shTRIM28 population [51]. We also captured the amplification of *SOX17* (endoderm), but only in one out of three biological replicates. Due to high deviations, the graph for *SOX17* expression represents only one replicate. The result is not statistically significant, but it is interesting enough to mention it in this study.

Until this point, our results suggested that TRIM28 phosphorylation does not significantly affect the mechanisms contributing to pluripotency and self-renewal maintenance in human iPSC. However, in the 3YF population, we noticed that the expression level of some differentiation markers (Figure 5D) was lower than in WT cells. This may support findings suggesting the inhibition of triple tyrosine phosphorylation impact on HP1BD interaction with HP1, which enables transcription repression by TRIM28 [19,52,53].

Still, our results imply that mutations of Ser473 or Ser824 do not affect TRIM28 function in human iPSC. These data stand in opposition to previous reports indicating the impact of Ser473 and Ser824 phosphorylation on TRIM28 function [18,31,43,44]. What is important, mentioned reports did not include the research on human iPSC. Nevertheless, the phosphorylation-dependent regulation of genes responsible for sustaining the undifferentiated state cannot be completely ruled out. Obtained data, however, indicates a much stronger influence of structural mutations on the mechanisms supporting pluripotency. Thus, only the populations with mutants in RING (C91A) or PHD (C628R) and shTRIM28 were subjected to further experiments.

### 3.9. Impairment of RING and PHD Functions Results in Dysregulation of Stem Cell-Associated Signaling Pathways

**Figure 6:**
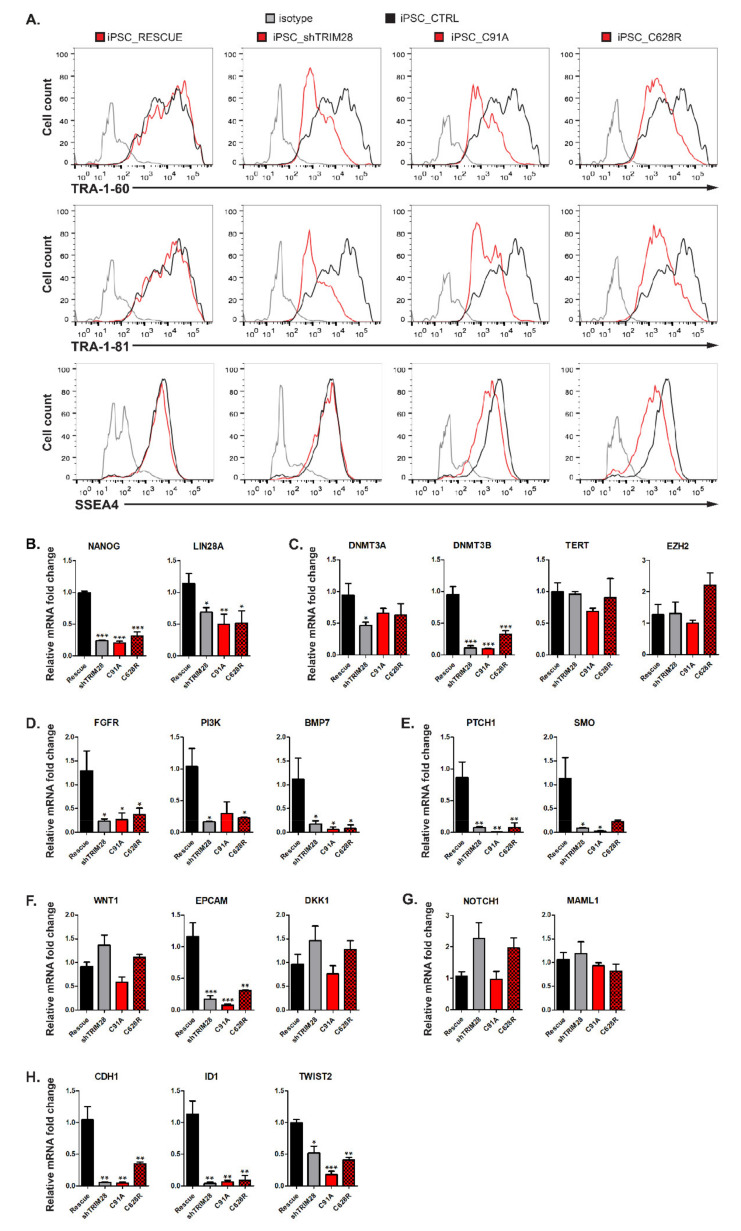
Impairment of RING and PHD functions results in dysregulation of stem cell-associated signaling pathways. (**A**) Extracellular pluripotency marker changes evaluated by flow cytometry. Fluorescence intensity for analyzed populations was compared to CTRL population and isotype control (*n* = 3). Representative histograms are shown for each population. (**B**–**H**) QRT-PCR analysis of genes dysregulated in differentiating populations. Graphs represent markers related to pluripotency (**B**), epigenetic regulation (**C**), self-renewal (**D**), Hedgehog pathway (**E**), WNT pathway (**F**), NOTCH pathway (**G**), migration, and metastasis (**H**). Each bar represents the mean mRNA expression level (SD), *n* = 3. Statistical analysis performed with one-way ANOVA and a post-hoc Dunnett’s test; *p* ≤ 0.05 (0.0001–0.001 ***; 0.001–0.01 **; 0.01–0.05 *; ≥0.05 not significant).

The differentiating shTRIM28, C19A, and C628R populations exhibited downregulation of extracellular pluripotency markers TRA-1-60 and TRA-1-81 (Figure 6A). The decreased expression of surface markers is more evident in cells with silenced *TRIM28* (shTRIM28) than in cells with a structural defect of RING or PHD domain. The SSEA-4 level was only slightly reduced in C19A and C628R mutants. However, the literature data indicate that the most rapid changes occur in the expression of TRA-1-81 and TRA-1-60 antigens, and the expression of SSEA-4 decreases much slower during differentiation of hESC [54]. QRT-PCR analysis of differentiating populations also showed significant downregulation of pluripotency markers *NANOG* and *LIN28A* (Figure 6B).

We next analyzed the expression of various markers implicated in the maintenance of self-renewal and stemness (Figure 6C–H). These genes are important for the functioning of both pluripotent and cancer cells. First, we investigated the expression of the genes engaged in chromatin modification (Figure 6C). Analyzed populations displayed downregulation of DNA methyltransferases (*DNMT*) 3A and 3B, which contributes to pluripotency maintenance and inhibition of differentiation [55,56]. Many reports indicate rapid repression of *TERT* within a few days upon differentiation [57,58]. However, according to our observations, TRIM28 RING and PHD domain dysfunctions do not seem to affect *TERT* expression level. *EZH2*, a set-domain containing histone methyltransferase specific to H3K27 [59], was upregulated only in the PHD-mutated population. However, this shift cannot be compared to the expression level in control or silenced *TRIM28* populations, and the results remain inconclusive.

Self-renewal regulation also depends on several signaling pathways, such as Wingless (Wnt) [60], Hedgehog (Hh) [61], phosphoinositide 3-kinase (Pi3K)/Akt kinase [5], or mitogen-activated protein kinases (MAPK) [62]. Therefore, we analyzed the expression of critical genes involved in these pathways. We observed that the downregulation of FGF receptor (*FGFR*), *PI3K*, and *BMP7* mRNA levels correlated with the impairment of RING and PHD function and the silencing of *TRIM28* (Figure 6D). The expression of key genes involved in the Hh pathway, receptor patched 1 (*PTCH1*) and smoothened (*SMO*), was also remarkably reduced in iPSC populations with silenced *TRIM28*, and in cells with RING and PHD mutants, indicating the inhibition of Hh signal transduction (Figure 6E). Structural mutations of *TRIM28* resulted in a significant decrease in the expression of *EPCAM*, pluripotency, and proliferation marker in mouse and human stem cells [63,64,65] (Figure 6F). Populations with silenced *TRIM28* and the population with PHD mutant showed a slight increase in *WNT* expression and downstream WNT signaling inhibitor *DKK1* [66]. Silencing of *TRIM28* and defects within the PHD contributed to a double increase in *NOTCH1* (Figure 6G). The expression of *MAML1*, which acts as a transcriptional coactivator for NOTCH signaling [67,68], did not alter under the influence of TRIM28 dysfunction. However, due to high deviations, the results for *WNT*, *DKK1*, *NOTCH1*, and *MAML1* expression were not considered statistically significant.

Finally, we examined the expression of genes that are known to be associated with migration and metastasis of cancer cells, and we observed a significant reduction in E-cadherin (*CDH1*), an inhibitor of differentiation 1 (*ID1*) and *TWIST2* (Figure 6H). We assumed that *CDH1* shift is directly related to the loss of typical compact colony morphology, regularly maintained in an undifferentiated state by cell-cell contact via E-cadherin [69,70]. Altogether, our results demonstrated that TRIM28 affects the expression of the genes implicated in the pathways common for stem cells and cancer cells.

## 4. Discussion

The TRIM28 protein was shown to have a crucial impact on self-renewal ability and maintenance of pluripotency in mouse and human ESC [8,16,17,18,71,72], as well as in human cancer cells [9,19,43]. In this study, we confirmed that TRIM28 preserves stemness and self-renewal in human iPSC. In addition, for the first time in human iPSC, we analyzed the function of individual TRIM28 domains. We identified RING and PHD as the principal domains responsible for these TRIM28 properties.

Impairment of RING or PHD activity, as well as *TRIM28* silencing, had a very rapid influence on iPSC populations. IPSC dissociated into individual cells, lost a typical compact colony structure, and showed a decrease in NANOG and SOX2 protein level. In contrast to previous studies suggesting the impact of Ser473 and Ser824 phosphorylation on TRIM28 function, examined phospho-mutants and phospho-mimetics did not affect the self-renewal in human iPSC [18,31,43,44]. Nevertheless, the inhibition of triple tyrosine phosphorylation (Y449F/Y458F/Y517F) slightly reduced the occasional differentiation of the 3YF population. This observation may support reports indicating 3YF mutation enables HP1BD interaction with HP1, resulting in transcription repression by TRIM28 [19,52,53]. Therefore, TRIM28 phosphorylation-dependent regulation of genes responsible for sustaining the undifferentiated state cannot be excluded. However, our data indicate a much stronger influence of structural mutations on the mechanisms supporting pluripotency.

Both mutations with observed effects on stemness properties (C91A, C628R) were predicted to have a high functional impact in our initial analysis with the Mutation Assessor algorithms. Indeed, our results underline the importance of both substituted amino acids for early developmental processes associated with the maintenance of self-renewal and pluripotency. Therefore, only the iPSC populations exhibiting phenotype changes were selected for further study.

Silencing *TRIM28* and impairment of RING or PHD domains activity led to downregulation of intra- and extra-cellular pluripotency markers. It also resulted in downregulation of methyltransferases *DNMT3A* and *DNMT3B*, associated with the ongoing switch in gene expression profile, progressive cell differentiation, and loss of the parental phenotype [55,56]. Our observations regarding RING domain function in stemness maintenance support the results indicating the engagement of KRAB-ZFPs in pluripotency maintenance. Silencing particular KRAB-ZFPs was shown to induce differentiation of pluripotent stem cells by epigenetic repression of crucial differentiation genes [8]. This may conclude that the RING domain, which is responsible for interaction with the KRAB domain of ZFPs, maintains self-renewal and stemness in human pluripotent stem cells through mediating KRAB-ZFPs repression function. On the other hand, we report that stemness maintenance in human iPSC is also determined by the activity of PHD, which is responsible for sumoylation, and therefore activation of the BROMO domain. Cooperation of PHD and BROMO domains results in chromatin remodeling, histone deacetylation, enhanced methylation of H3K9, and finally binding HP1 to tri-methylated H3K9 and silencing gene expression [30,73]. Our data indicate that TRIM28, to support pluripotency and self-renewal mechanisms, requires functional RBCC and PHD domains that are responsible for the activity of TRIM28 as a transcriptional co-repressor. Gene repression mediated by TRIM28 is also compromised by phosphorylation of S824 TRIM28 residue. In opposition to previous data indicating the important role of this post-translational modification in maintaining pluripotency in murine cells [18], S824A phospho-mutant was shown to be insufficient to initiate differentiation in human iPSC in our study.

In mouse ESC, Trim28, along with Cnot3 co-repressor, were identified as factors necessary to maintain self-renewal capacity [71]. They were shown to bind to numerous gene promoters, creating a unique module distinct from the main module of the core pluripotency network, formed by *OCT3/4*, *SOX2*, and *NANOG*. The sequences regulated by TRIM28 included genes involved in the cell cycle, cell death, and tumorigenesis [71]. Here, we show that during differentiation of iPSC due to TRIM28 depletion, there is a significant shift in cellular signaling. We demonstrate that silencing *TRIM28* leads to upregulation of processes related to the regulation of apoptosis, differentiation into a multicellular organism, positive regulation of the developmental process, and regulation of the cell cycle, which is also confirmed by earlier evidence [9,74,75].

As previously reported, TRIM28 overexpression correlates with poor prognosis in many cancer types [20,21,22,23,24,25]. Furthermore, there is growing evidence that cancer stemness is associated with the expression of *OCT3/4*, *SOX2*, *c-MYC*, and other genes involved in the self-renewal regulation of malignant cancer cells, as well as normal stem cells [3]. Numerous studies have been performed to characterize tumor cells in terms of their stemness score and similarity to normal stem cells. Genes upregulated in ESC, for instance genes regulated by NANOG, OCT3/4, SOX2, and c-MYC, are often overexpressed in undifferentiated tumors compared to differentiated ones [76,77,78]. Collectively, with many reports presenting TRIM28 contribution to cancer, these findings suggest that regulatory networks controlling self-renewal in stem cells may also be active in some types of cancer and may constitute new cancer cell therapy.

Our results also indicate that the dysfunction of RING or PHD domains decreases proliferation and EBs formation of hiPSC, which might be very important considering TRIM28 correlation with some tumors. For this reason, we also analyzed the expression of various markers involved in the maintenance of self-renewal, stemness, and the functioning of pluripotent stem cells, as well as of highly dedifferentiated cancer cells.

In human ESC, the pivotal factor in maintaining the state of pluripotency is FGF, which activates PI3K/Akt and MAPK/Erk signaling cascades [62]. Our results reveal a significant reduction in *FGFR2* and *PI3K* expression in all differentiating iPSC populations. PI3K/Akt signaling maintains pluripotency by regulating OCT4, SOX2, and NANOG [79,80]. Zhou et al. demonstrated that in hESC inhibition of mTOR, which is a PI3K effector, this results in a decrease in OCT4, SOX2, and NANOG expression [79]. On the contrary, our results indicate that the most significant changes in the expression of core pluripotency transcription factors occurring within the timescale of our experiment are limited to *NANOG*. Thus, our data may suggest that the differentiation effect was caused not by the primary inhibition of FGF/PI3K signaling but by the lower *NANOG* expression caused by disrupted TRIM28 activity.

Recently Do et al. determined that Trim28 prevents the degradation of Oct4, and Trim28 overexpression stabilizes Oct4 in mouse ESC [81]. Furthermore, they found the Trim28 CC domain to be responsible for interaction with Oct4. This supports our findings, indicating that only silencing of *TRIM28* resulted in downregulation of OCT4 expression, and none of the introduced mutations were able to induce such an effect. Data presented by Do et al. may also support reports regarding TRIM28 overexpression correlation with poor prognosis in certain cancer types [20,21,22,23,24,25].

We also demonstrate that dysfunction of the RING or PHD domains and *TRIM28* silencing leads to the downregulation of the Hedgehog pathway and *EPCAM*, implicated in stemness maintenance in iPSC. These factors are also therapeutic targets for cancer. In adults, the mutation or deregulation of the Hedgehog pathway plays a key role in both proliferation and differentiation, leading to tumorigenesis or accelerated tumor growth in many different tissues [4,82,83]. *EPCAM* is frequently overexpressed in tumor cells [84], while its suppression is considered a new approach for the treatment of colon cancer [85]. Furthermore, we show that TRIM28 significantly impacts the expression of metastasis-related genes, *TWIST2* and *ID-1*. Many reports indicate the involvement of TRIM28 in the induction of EMT through regulation of *TWIST* [9,86,87]. ID-1 contributes to many cellular processes, including cell growth, aging, differentiation, apoptosis, angiogenesis, and neoplastic transformation [88,89,90]. Recent studies suggest that *ID-1* knock-down in endothelial cells derived from angioma inhibits proliferation and induces apoptosis by inhibiting PI3K/Akt/mTOR signaling [90]. Our data also indicate a significant reduction in *ID-1* expression, as well as a decrease in *PI3K* activity in cells with impaired TRIM28 functions, which supports previous findings.

Although both RING and PHD domains contribute to the self-renewal and stemness of human iPSC, they may use different interactions and mechanisms to repress gene expression. We found that *WNT1*, *DKK1*, and *NOTCH1* showed a trend of increased expression in the population with mutated PHD compared to RESCUE and RING mutants. The expression level of *WNT1*, *DKK1*, and *NOTCH1* in the PHD mutant population is similar to the expression in cells with silenced *TRIM28*. In stem cells, the activation of Wnt signaling can induce the expression of Notch pathway components [91]. This may suggest that in contrast to the RING domain, PHD takes part in Wnt/Notch signaling.

## 5. Conclusions

In conclusion, our study provides new insights into the role of TRIM28 protein domains in the regulation of pluripotency processes and self-renewal mechanisms in human induced pluripotent stem cells. Of the numerous biological functions of TRIM28 protein, our results indicate the activity of RING and PHD domains in the transcriptional repression to be one of the main molecular mechanisms responsible for maintaining self-renewal and pluripotency. In addition, we demonstrate that in iPSC, TRIM28 influences the expression of the genes involved in the cell cycle, self-renewal, cell death, mobility, and other gene characteristic for cancer cells. Therefore, regulatory networks dependent on TRIM28 signaling that control self-renewal in stem cells may also be active in some types of cancer cells. As such, TRIM28 RING and PHD domains may be considered new targets for cancer therapy, e.g., by designing a drug inhibitor complementary to selected domains. The application of an oncolytic virus expressing a short peptide, binding to crucial TRIM28 amino acids and blocking domain interactions, could also prove useful.

## Data Availability

The RPPA data that support the findings of this study are openly available in GEO at www.ncbi.nlm.nih.gov/geo (accessed on 27 June 2021), reference number GSE153726. The data that support the findings of this study are available from the corresponding author upon request.

## Supplementary Materials

The following are available online at https://www.mdpi.com/article/10.3390/cells10081933/s1, Figure S1: TRIM28 protein modulates the structure of chromatin, Figure S2: generating human iPSC with doxycycline-inducible system results in repression of transgene expression in established clones, Figure S3: proteomic profile of iPSC with silenced TRIM28 indicates upregulation of apoptotic and differentiation processes, Table S1: the structure and interactions of TRIM28 protein, Table S2: a list of oligonucleotides used in the study, Table S3: a list of antibodies used in the study, Video S1: WT, Video S2: CTRL, Video S3: RESCUE, Video S4: shTRIM28, Video S5: S473A, Video S6: S473D, Video S7: S824A, Video S8: S824D, Video S9: 3YF, Video S10: C91A, Video S11: C628R, Video S12: N773G.

## Abbreviations

BROMO	Bromodomain
CC	Coiled-Coil Motif
EB	Embryoid Bodies
ESC	Embryonic Stem Cells
FGFR	Fibroblast Growth Factor Receptor
GSEA	Gene Set Enrichment Analysis
HP1BD	Heterochromatin Protein 1-Binding Domain
iPSC	induced Pluripotent Stem Cells
KAP1	Krüppel-Associated Box-Associated Protein 1
KRAB-ZFPs	KRAB Zinc-Finger Proteins
LV	*Lentivirus*
MEF	Mouse Embryonic Fibroblasts
NuRD	Nucleosome Remodeling and Deacetylation Complex
ORA	Over-Representation Enrichment Analysis
OXPHOS	Oxidative Phosphorylation
PCA	Principal Component Analysis
PHD	Plant Homeodomain
PHDF	Primary Human Dermal Fibroblasts
RBCC	RING-B-box-Coiled-Coil domain
RING	Really Interesting New Gene
RPPA	Reverse Phase Protein Array
SETDB1	SET Domain Bifurcated 1 Histone Lysine Methyltransferase
shRNA	small hairpin RNA
siRNA	small interfering RNA
TIF1β	Transcription Intermediary Factor 1-β
TRIM	Tripartite Motif
TRIM28	Tripartite Motif-Containing Protein 28
