# Supplementary material for: Disruption of RING and PHD Domains of TRIM28 Evokes Differentiation in Human iPSCs
Source: Cells. 2021 Jul 29;10(8):1933. doi: 10.3390/cells10081933 (PMC8394524; doi:10.3390/cells10081933)
Supplement: Supplementary file 1 [file cells-10-01933-s001.zip › cells-1286951-supplementary resubmitted/Supplementary Figures legend.pdf]

Figure S1. TRIM28 protein modulates the structure of chromatin.

A. The architecture and strictly defined functions of the TRIM28 protein domains.
B. The interactions of TRIM28 with multiple proteins, required for its transcription repression activity.

Figure S2. Generating human iPSC with doxycycline-inducible system results in repression of transgene expression in established clones

A. Doxycycline inducible system of transgene expression was used to dedifferentiate PHDF cells into iPSC and switch off transgene expression.
B. Morphology changes of reprogrammed PHDF cells after LV-Stemcca-TetO transduction. Scale bar 100 μm. Single iPSC colonies were manually picked and propagated in further clonal culture. On day 21, cells were transduced with LV carrying tTRKRAB sequence inhibiting Stemcca-TetO transgene expression.
C. Pluripotency analysis of obtained iPSC lines by immunofluorescence staining against intra- (OCT3/4, NANOG) and extracellular pluripotency markers (SSEA-4, TRA-1-60, TRA-1-81), and differentiation marker SSEA1 (red, green – analyzed genes; blue – DAPI). Scale bar 100 μm, SSEA4 – 200 μm.
D. Bisulfite genomic sequencing analysis of methylation status of CpG in the promoter regions of OCT3/4 and NANOG in generated iPSC, compared to methylation status in hESC (positive control) and in PHDF cells (negative control). The image shows representative data for indicated cell lines. White circles – no CpG methylation, black circles – CpG methylation.
E. RT-PCR analysis of mRNA level of pluripotency markers in obtained iPSC lines was compared to expression in hESC as a positive control and expression in PHDF cells (negative control).
F. Representative chromosomal G-band analysis of generated iPSC lines confirming their normal karyotype.
G. Immunohistochemical staining of excised teratomas (upper panel) confirming iPSC potential to differentiate into ecto-, endo-, and mesoderm. Histological analysis of H+E stained teratomas (lower panel) revealed presence of structures characteristic for mesoderm (cartilage), endoderm (glandular tissue) and ectoderm (neuroepithelium). The image shows representative data for indicated cell lines.
H. RT-PCR analysis of mRNA in the material isolated from iPSC-derived teratomas with primers specific for the Stemcca-TetO transgene. The mRNA level was compared to the expression in PHDF cells transduced only with LV-Stemcca-TetO, without LV-tTRKRAB (positive control), and in unmodified PHDF cells (negative control).

Figure S3. Proteomic profile of iPSC with silenced *TRIM28* indicates upregulation of apoptotic and differentiation processes

A-B. The list of upregulated markers assigned to individual Gene Ontology Biological Processes significantly enriched in **(A.)** iPSC-siTRIM28 relative to reference iPSC and **(B.)** reference iPSC relative to iPSC-siTRIM28 as determined with Overrepresentation Enrichment Analysis (ORA) using WebGestalt on-line tool. Markers are arranged in order of protein level. The relative protein level available in the heatmap in Figure S2E and S2F. Top 25 terms with FDR < 0.05% are presented.
