# Supplementary material for: Disruption of RING and PHD Domains of TRIM28 Evokes Differentiation in Human iPSCs
Source: Cells. 2021 Jul 29;10(8):1933. doi: 10.3390/cells10081933 (PMC8394524; doi:10.3390/cells10081933)
Supplement: Supplementary file 1 [file cells-10-01933-s001.zip › cells-1286951-supplementary resubmitted/Table S1.pdf]

Table S1. The structure and interactions of TRIM28 protein

TRIM28 domain	Domain subunit	Key subunit features	Interactions of domain	Consequences of interaction
RBCC	RING	Binds two zinc ions of zinc-finger proteins (ZFPs) [11].<br><br>Has properties of E3 ubiquitin ligase [11].<br><br>Defects in RING E3 ubiquitin ligases are associated with tumor development, and part of the RING E3 ligases is overexpressed in cancer [32].	RBCC is indispensable and sufficient to bind with high affinity to the KRAB domain of KRAB-ZFPs [11].<br><br>KRAB domain, for its repressor activity, requires binding to the co-repressor, TRIM28 [33].<br><br>RBCC-KRAB interaction requires RBCC oligomerization as a homotrimer.<br>Mutation of any subdomain ablates the oligomerization and binding ability to the KRAB domain [33,34].	TRIM28/KRAB-ZFPs modify DNA methylation patterns resulting in reversible [35] and irreversible [36] gene repression.
	B-Box	Folds independently and binds a single zinc ion [33].		
	CC	The structure of coiled-coil alpha-helices [11].<br><br>Necessary for a protein-protein interaction via the RBCC domain [11].<br>Prevents Oct4 from degradation in mouse ESC [37].		
HP1-BD	-	Includes hydrophobic PxVxL pentapeptide sequence [31].	Recruits HP1 protein. Interaction is necessary to silence DNA sequence [38].	HP1 directly binds with high affinity to H3K9me2/3, constituting a transcriptionally inactive chromatin marker, and maintains this state [38,39].
PHD-BROMO functional unit	PHD	Has E3 ligase activity and sumoylates the adjacent bromodomain [28].	Sumoylated bromodomain binds with SETDB1 and with SUMO interaction domains (SIM) in the Mi2/NuRD complex [28,40].	SETDB1 methyltransferase is specific for histone H3 lysine 9 (H3K9). The resulting H3K9me3 stimulates the binding of HP1 protein to histone H3 [30].<br><br>NuRD complex subunits, histone deacetylase 1 and 2 (HDAC1/2), modify histone tails.<br>The subunit Mi2 enables chromatin remodeling (ATP-helicase activity) [41].
	BROMO	Its SUMO modification has a major role in TRIM28 repression activity [29].		
