# Supplementary material for: Disruption of RING and PHD Domains of TRIM28 Evokes Differentiation in Human iPSCs
Source: Cells. 2021 Jul 29;10(8):1933. doi: 10.3390/cells10081933 (PMC8394524; doi:10.3390/cells10081933)
Supplement: Supplementary file 1 [file cells-10-01933-s001.zip › cells-1286951-supplementary resubmitted/Table S2.pdf]

Table S2. A list of oligonucleotides used in the study

	Oligonucleotide	Sequence 5′ -> 3′
qRT-PCR primers	B2M_F	TTCTGGCCTGGAGGCTATC
	B2M_R	TCAGGAAATTTGACTTTCCATTC
	BMP7_F	ACCACTGGGTGGTCAATCC
	BMP7_R	CAACTTGGGGTTGATGCTCT
	Brachyury_F	ATGATGGAGGAACCCGGAGA
	Brachyury_R	TGGTTCCAGGAAGAAGCCAC
	CDH1_F	CCCGGGACAACGTTTATTAC
	CDH1_R	GCTGGCTCAAGTCAAAGTCC
	CDX2_F	CCCACAGCCATAGACCTACAG
	CDX2_R	CCCCAAATCCCACTTGTCT
	DKK1_F	CAGGCGTGCAAATCTGTCT
	DKK1_R	AATGATTTTGATCAGAAGACACACATA
	DNMT3A_F	CCACTGTGAATGATAAGCTGGA
	DNMT3A_R	AATGGTCCTCACTTTGCTGAA
	DNMT3B_F	GGAAATTAGAATCAAGGAAATAC
	DNMT3B_R	AATTTGTCTTGAGGCGCTTG
	EPCAM_F	CCATGTGCTGGTGTGTGAA
	EPCAM_R	TGTGTTTTAGTTCAATGATGATCCA
	EZH2_F	TGTGGATACTCCTCCAAGGAA
	EZH2_R	GAGGAGCCGTCCTTTTTCA
	FGFR_F	CCAAAATGGGAGTTTCCAAG
	FGFR_R	CATGACCACTTGCCCAAAG
	FSP1_F	TCAGAACTAAAGGAGCTGCTGA
	FSP1_R	TTGGAAGTCCACCTCGTTGT
	GABRB3_F	CCTTGCCCAAAATCCCCTATGTCAAAGC
	GABRB3_R	GTATCGCCAATGCCGCCTGAGACCTC
	GAPDH_F	AGCCACATCGCTCAGACAC
	GAPDH_R	GCCCAATACGACCAAATCC
	GATA4_F	TGCGCGGAACCTCCAG
	GATA4_R	AGAAGCTGCTAGGCTGGCTC
	GATA6_F	CGGGCTCTACAGCAAGATGA
	GATA6_R	TGACAGTTGGCACAGGACAA
	GFAP_F	AGCTCCAGGATGAAACCAAC
	GFAP_R	GGCTTCATCTGCTTCCTGTC
	HPRT1_F	TGACCTTGATTTATTTTGCATACC
	HPRT1_R	CGAGCAAGACGTTCAGTCCT
	ID1_F	CGACATGAACGGCTGTTACTC
	ID1_R	CTCACCTTGCGGTTCTGG
	LIN28A_F	GAAGAAGAAATCCACAGCCCTA
	LIN28A_R	AGAATAGCCCCCACCCATT
	MAML1_F	CACCCAGGCCTACCCTGT
	MAML1_R	CTGCCCGCTATAGGCAAA
	MAP2_F	CGAACTTTATATTTTACCACTTCCTTG
	MAP2_R	CCGTTCATCTGCCATTCTTC
	MSX1_F	CTCGTCAAAGCCGAGAGC
	MSX1_R	CGGTTCGTCTTGTGTTTGC
	NANOG_F	AGATGCCTCACACGGAGACT
	NANOG_R	TTTGCGACACTCTTCTCTGC
	Nestin_F	TGCGGGCTACTGAAAAGTTC
	Nestin_R	GAGCGATCTGGCTCTGTAGG

	NODAL_F	GGGCAAGAGGCACCGTCGACATCA
	NODAL_R	GGGACTCGGTGGGGCTGGTAACGTTTC
	NOTCH1_F	AGGCAATCCGAGGACTATGA
	NOTCH1_R	GCTCAGAACGCACTCGTTG
	OCT3/4_F	GACAGGGGGAGGGGAGGAGCTAGG
	OCT3/4_R	CTTCCCTCCAACCAGTTGCCCCAAAC
	PAX6_F	TGCTCCGGCATGAAATATACTA
	PAX6_R	GTCTCCAAATGTGCAGCAAC
	PIK3_F	CTCTCCTGTGCTGGCTACTGT
	PIK3_R	GCTCTCGGTTGATTCCAAACT
	PTCH1_F	CATGTTTGCACCCGTCCT
	PTCH1_R	CCAGCACAGCAAAGAAATACC
	REX1_F	CAGATCCTAAACAGCTCGCAGAAT
	REX1_R	GCGTACGCAAATTAAAGTCCAGA
	RPLP0_F	TCTACAACCCTGAAGTGCTTGAT
	RPLP0_R	CAATCTGCAGACAGACACTGG
	SMA_F	CCTATCCCCGGGACTAAGACG
	SMA_R	CTTACAGAGCCCAGAGCCAT
	SMO_F	ACCCCAAACCCATCTTTTGT
	SMO_R	CACCGCTGAAACTGAACTGA
	SOX1_F	ACCAGGCCATGGATGAAG
	SOX1_R	CTTAATTGCTGGGGAATTGG
	SOX17_F	AACGCCGAGTTGAGCAAGA
	SOX17_R	GGGGTGGTCCTGCATGTG
	TERT_F	GGGCTGAGTGTCCAGCAC
	TERT_R	CTCCTGGTGAGGAAAAGCT
	Transgene_F	TGGGAGCCCTCACTTCAC
	Transgene_R	AACGCGAACGTGGAGAAAG
	TRIM28_F	CAGCTGGCTACCGACTCC
	TRIM28_R	GCACGGATCAGGGTCAGA
	TUJ_F	GCGGATCAGCGTCTACTACA
	TUJ_R	GGCCTGAAGAGATGTCCAAA
	TWIST2_F	GCAAGAAGTCGAGCGAAGAT
	TWIST2_R	GCTCTGCAGCTCCTCGAA
	WNT1_F	CGAGTGCAAGTGGCAGTTC
	WNT1_R	AACGCCGTTTCTCGACAG
	WPRE_F	GTCCTTTCCATGGCTGCTC
	WPRE_R	CCGAAGGGACGTAGCAGA
Site directed mutagenesis primers (introduced mutations underlined)	RING_F	TCGGCCTGTAGTGCCGCTTTAGGGCCCGCGGCC
	RING_R	GGCCGCGGGCCCTAAAGCGGCACTACAGGCCGA
	PHD_F	GACAGTGCCACCATTCGCCGTGTCTGCCAGAAG
	PHD_R	CTTCTGGCAGACACGGCGAATGGTGGCACTGTC
	BROMO_F	ATGTTCAAGCAATTCGGTAAGTTAACTGAGGAC
	BROMO_R	GTCCTCAGTTAACTTACCGAATTGCTTGAACAT
TRIM28 sequencing primers with TRIM28 hybridisation location	-113 nt (PAC/2A sequence)	GTGCATGACCCGCAAGCC
	245-265 nt	CTGTTTGCACTCGGCCTGTA
	568-588 nt	ATACTGTGCGCTCTACTGGG
	1032-1052 nt	ATTCTGCGCTTTGCCTCTTG
	1509-1529 nt	CCCGTCTTCAAGGTCTTCCC
	1974-1993 nt	GTACCAGGGGAGGAGTGGA
siRNA targeting endogenous TRIM28 sequence	siRNA_TRIM28 sense	CGACUAGCUGUGAGGAUAATT
	siRNA_TRIM28 antisense	UUAUCCUCACAGCUAGUGCTT
	siRNA_TRIM28_validated sense	CGUGUACUGCUGGCCCUAUTT

	siRNA_TRIM28_validated antisense	AUAGGGCCAGCAGUACACGTT
Promoter amplification primers	bsOCT3/4_F	GGATGTTATTAAGATGAAGATAGTTGG
	bsOCT3/4_R	CCTAAACTCCCCTTCAAAATCTATT
	NANOG_F	TTAAAAATTAAGAAAAAGGT
	NANOG_R	AACTAATTTCAAACTCCTAACTTCAA
Promoter sequencing primer	T7 sequencing primer	TAATACGACTCACTATAGGG
