# Supplementary material for: Disruption of RING and PHD Domains of TRIM28 Evokes Differentiation in Human iPSCs
Source: Cells. 2021 Jul 29;10(8):1933. doi: 10.3390/cells10081933 (PMC8394524; doi:10.3390/cells10081933)
Supplement: Supplementary file 1 [file cells-10-01933-s001.zip › cells-1286951-supplementary resubmitted/Table S3.pdf]

Table S3. A list of antibodies used in the study

Antibody	Source	Identifier	Application
Anti-Nanog, Mouse Monoclonal	Thermo Fisher Scientific, Waltham, MA, USA	Cat# 14-5769-82; RRID:AB_467574	Immunofluorescence
Anti-Oct4, Rabbit Polyclonal	Abcam, Cambridge, GB	Cat# ab18976; RRID:AB_444714	Immunofluorescence
Anti-Oct4, Mouse Monoclonal	Abcam, Cambridge, GB	Cat# ab184665	Immunofluorescence
Anti-SOX2, Rabbit Polyclonal	Abcam, Cambridge, GB	Cat# ab97959; RRID:AB_2341193	Immunofluorescence
Anti TRIM28, Mouse Monoclonal	Cell Signaling Technology, Danvers, MA, USA	Cat# 5868; RRID:AB_10707324	Immunofluorescence
Anti TRIM28, Rabbit Monoclonal	Cell Signaling Technology, Danvers, MA, USA	Cat# 4124; RRID:AB_2209886	Immunofluorescence
Anti-FLAG, Mouse Monoclonal	Sigma-Aldrich, St, Louis, MO, USA	Cat# F3165; RRID:AB_259529	Immunofluorescence
Anti-SOX17, Rabbit Polyclonal	Millipore Merck KGaA, Darmstadt, Germany	Cat# 09-038; RRID:AB_1587525	Immunofluorescence
Anti-ACTA2, Mouse Monoclonal	Thermo Fisher Scientific, Waltham, MA, USA	Cat# MA1-06110; RRID:AB_557419	Immunofluorescence
Anti-TUBB3, Rabbit Polyclonal	Abcam, Cambridge, GB	Cat# ab18207; RRID:AB_444319	Immunofluorescence
Anti-SSEA4, Mouse Monoclonal	Thermo Fisher Scientific, Waltham, MA, USA	Cat# 14-8843-80; RRID:AB_657847	Flow Cytometry
Anti-TRA-1-60, Mouse Monoclonal	Thermo Fisher Scientific, Waltham, MA, USA	Cat# 14-8863-82; RRID:AB_891610	Flow Cytometry
Anti-TRA-1-81, Mouse Monoclonal	Thermo Fisher Scientific, Waltham, MA, USA	Cat# 14-8883-82; RRID:AB_891614	Flow Cytometry
Anti-Mouse IgM Biotin, Rat Monoclonal	Thermo Fisher Scientific, Waltham, MA, USA	Cat# 13-5790-82; RRID:AB_466675	Flow Cytometry
Anti-Mouse IgG Biotin, Rat Monoclonal	Thermo Fisher Scientific, Waltham, MA, USA	Cat# 13-4013-85; RRID:AB_466650	Flow Cytometry
Anti-Mouse IgG H&L (Alexa Fluor® 594), Goat Polyclonal	Abcam, Cambridge, GB	Cat# ab150116; RRID:AB_2650601	Immunofluorescence
Anti-Mouse IgG H&L (Alexa Fluor® 488), Goat Polyclonal	Abcam, Cambridge, GB	Cat# ab150113; RRID:AB_2576208	Immunofluorescence
Anti-Rabbit IgG H&L (Alexa Fluor® 488), Goat Polyclonal	Abcam, Cambridge, GB	Cat# ab150077; RRID:AB_2630356	Immunofluorescence
Anti-Rabbit IgG H&L (Alexa Fluor® 594), Goat Polyclonal	Abcam, Cambridge, GB	Cat# ab150080; RRID:AB_2650602	Immunofluorescence
Anti-SSEA1, Mouse Monoclonal	R&D Systems, Minneapolis, MN, USA	Cat# MAB2155; RRID:AB_358058	Immunofluorescence
Anti-FOXA2, Rabbit Polyclonal	Millipore Merck KGaA, Darmstadt, Germany	Cat# AB4125; RRID:AB_2104889	Immunofluorescence
Anti-PAX6, Rabbit Polyclonal	Abcam, Cambridge, GB	Cat# ab5790; RRID:AB_305110	Immunofluorescence

anti-ATP5H, Mouse Monoclonal	Abcam, Cambridge, GB	Cat# ab110275; RRID:AB_10861517	Immunofluorescence
anti-PGK1, Rabbit Polyclonal	Abcam, Cambridge, GB	Cat# ab38007; RRID:AB_2161220	Immunofluorescence
anti-PKM2, Rabbit Polyclonal	Abcam, Cambridge, GB	Cat# ab85555; RRID:AB_10562282	Immunofluorescence
anti-HK1, Rabbit Polyclonal	Abcam, Cambridge, GB	Cat# ab65069; RRID:AB_1140844	Immunofluorescence
anti-HK2, Mouse Monoclonal	Abcam, Cambridge, GB	Cat# ab104836; RRID:AB_10710018	Immunofluorescence
anti-Beta Actin, Rabbit Polyclonal	Abcam, Cambridge, GB	Cat# ab75186; RRID:AB_1280759	Western Blot
anti-FLAG, Rabbit Monoclonal	Cell Signaling Technology, Danvers, MA, USA	Cat#14793; RRID:AB_2572291	Western Blot
anti-Rabbit IgG H&L (HRP), Goat Polyclonal	Abcam, Cambridge, GB	Cat# ab205718; RRID:AB_2819160	Western Blot
anti-Mouse IgG H&L (HRP), Goat Polyclonal	Abcam, Cambridge, GB	Cat# ab205719; RRID:AB_2755049	Western Blot
