# Supplementary figures and images for: Disruption of RING and PHD Domains of TRIM28 Evokes Differentiation in Human iPSCs
Source: Cells. 2021 Jul 29;10(8):1933. doi: 10.3390/cells10081933 (PMC8394524; doi:10.3390/cells10081933)

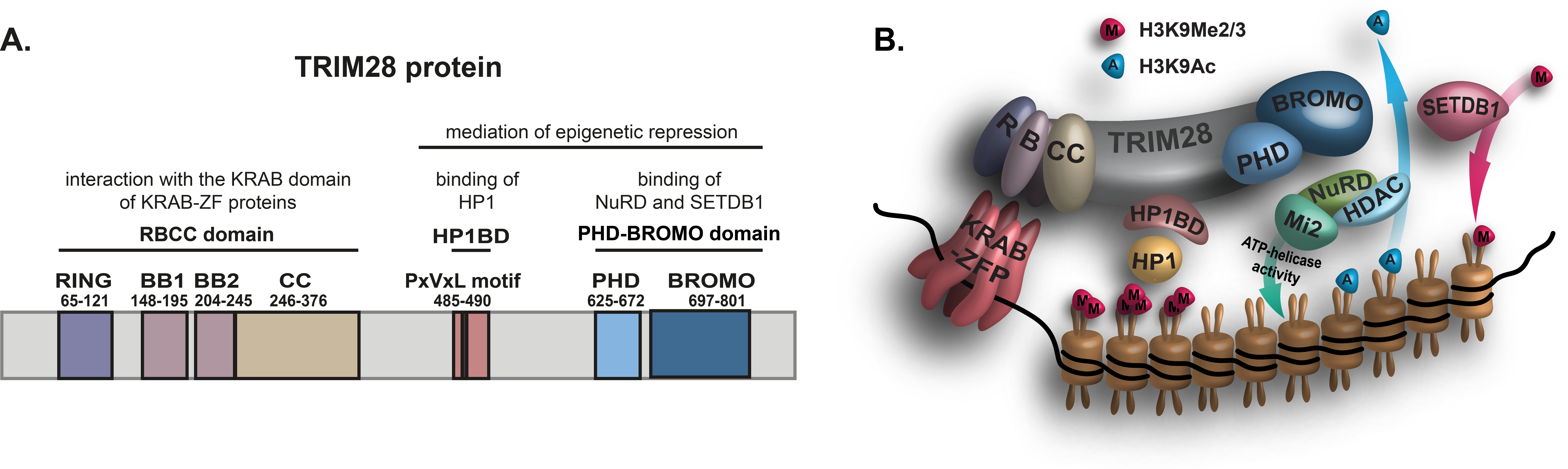

Supplement: Supplementary file 1 [file cells-10-01933-s001.zip › cells-1286951-supplementary resubmitted/Figure S1_TRIM28 protein modulates the structure of chromatin-01.tif]

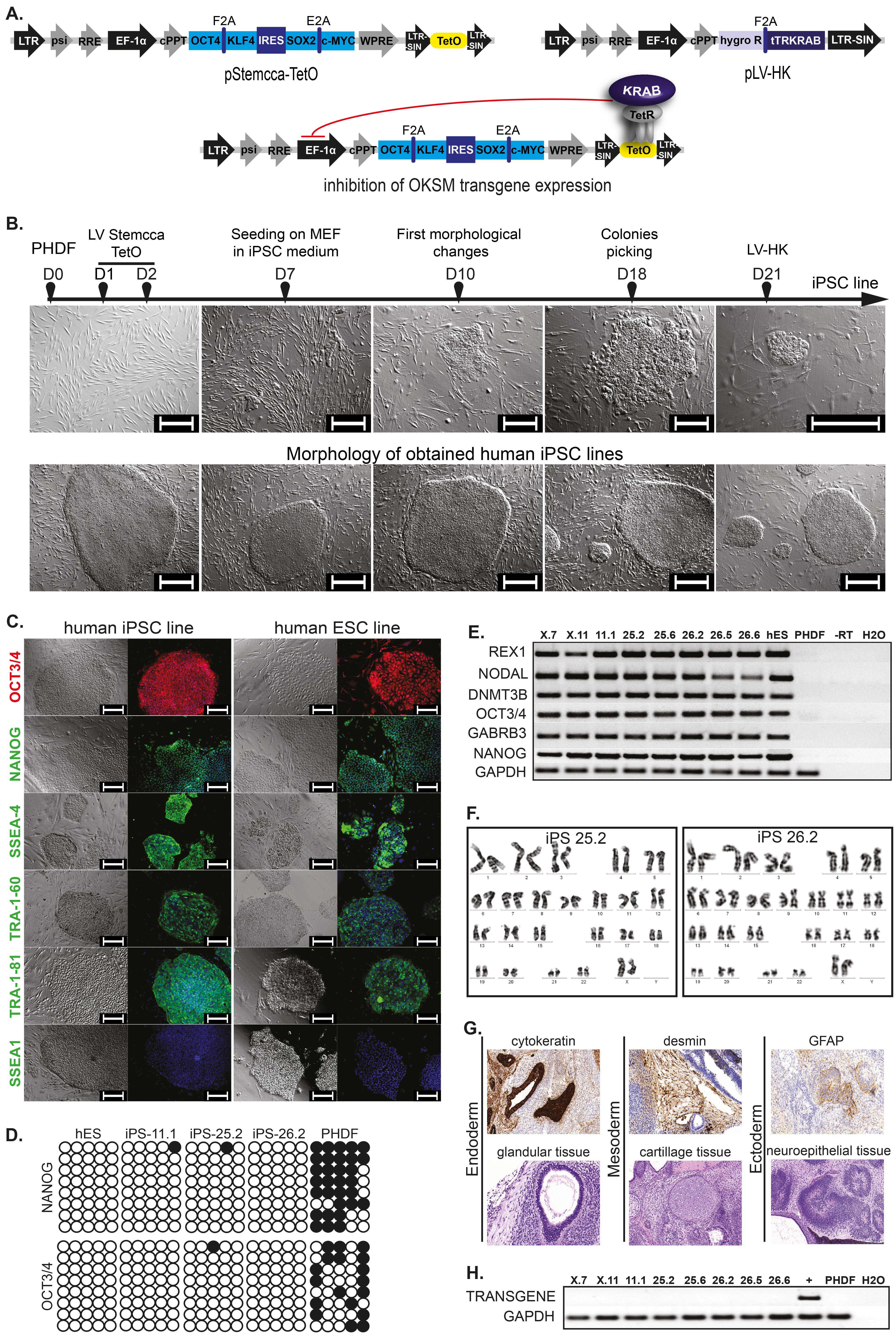

Supplement: Supplementary file 1 [file cells-10-01933-s001.zip › cells-1286951-supplementary resubmitted/Figure S2_Generating human iPSC with doxycycline-inducible system results in transgene repression in established clones-01.tif]
